# Medroxyprogesterone promotes neuronal survival after cerebral ischemic stroke by inhibiting PARthanatos

**DOI:** 10.3389/fphar.2025.1487436

**Published:** 2025-02-13

**Authors:** Chang-Ling Yue, Yan-Xia Ding, Meng Chen, Yu-Xin Shen, Ao-Meng Hu, Hai Huang, Zhao-Huan Zhang, Ying-Xin Zhou, Xiao-Hui Xu

**Affiliations:** ^1^ School of Life Sciences, Shanghai University, Shanghai, China; ^2^ School of Preclinical Medicine, Wannan Medical College, Wuhu, China; ^3^ Anhui Province Key Laboratory of Basic Research and Transformation of Age-related Diseases, Wannan Medical College, Wuhu, China; ^4^ School of Nursing, Henan University of Chinese Medicine, Zhengzhou, China; ^5^ Department of Laboratory Medicine, Changzheng Hospital, Naval Medical University, Shanghai, China

**Keywords:** medroxyprogesterone, PARthanatos, apoptosis-inducing factor, extracellular signal-regulated kinase, cerebral ischemic stroke, approved drug library

## Abstract

**Introduction:**

Cerebral ischemic stroke (CIS) is caused by the interruption of cerebral blood circulation due to thrombosis or embolism and is the second-leading cause of mortality worldwide. The neuronal death and motor dysfunction resulting from CIS are primarily attributed to the induction of PARthanatos in neurons at the site of ischemia. Blocking parthanatos is a promising treatment for CIS.

**Methods:**

The effect of medroxyprogesterone treatment on PARthanatos in vitro was examined by CCK8 assay and flow cytometry and the target protein of medroxyprogesterone was then identified by a series of assays, including western blotting, immunofluorescence, cell thermal shift assay and molecular docking. Subsequently, the efficacy of medroxyprogesterone in the treatment of ischemic stroke was evaluated by FJC staining.

**Results:**

In our study, medroxyprogesterone was able to block the occurrence of PARthanatos in Hela cells induced by MNNG. PARP-1 activity did not change after medroxyprogesterone treatment but prevented MNNG-induced apoptosis inducing factor (AIF) release from mitochondria by improving the stability of phosphorylated extracellular signal-regulated kinase (ERK). In vivo, medroxyprogesterone significantly reduces neuronal death in mouse models of CIS by inhibiting PARthanatos.

**Conclusion:**

Our findings indicate that medroxyprogesterone effectively inhibits PARthanatos not by affecting the activity of PARP-1, but by directly binding to ERK and stabilizes the active phosphorylated ERK, thereby inhibiting AIF translocation. Furthermore, medroxyprogesterone shows potential as a neuroprotective agent for patients with CIS, potentially enhancing post-stroke recovery and reducing societal burdens.

## 1 Instruction

Cerebral ischemic stroke (CIS) results from the disruption of cerebral blood flow and is the second-leading cause of mortality worldwide, accounting for 5.9 million deaths and 102 million disabilities annually (Qin et al., 2022). Following ischemia, ionic imbalance facilitates the excessive binding of the excitatory neurotransmitter glutamate to the NMDA receptor, leading to its activation. This activation results in an influx of calcium ions and the subsequent production of substantial amounts of nitric oxide (NO) and reactive oxygen species (ROS) (Liu et al., 2022). The generated NO reacts with superoxide to form excessive peroxynitrite (ONOO-), an unstable oxidant that induces DNA fragmentation and breakage. Consequently, this results in the overactivation of PARP-1, which contributes to the onset of PARthanatos in damaged neurons (Wang et al., 2019; Koehler et al., 2021). Currently, stroke management primarily involves prompt clinical interventions, such as intravenous thrombolysis and mechanical thrombectomy, to restore cerebral vessel patency, alongside antithrombotic therapies, including antiplatelet or anticoagulant treatments. Nonetheless, there is currently no available treatment to prevent neuronal cell death (Qin et al., 2022), highlighting the urgent necessity for the development of pharmacological agents aimed at inhibiting neuronal cell death.

Currently, PARP-1, a pivotal molecule associated with the PARthanatos pathway, is extensively regarded as a potential target for modulating and ameliorating neuronal cell injury (Koehler et al., 2021; Liu et al., 2022). Although the inhibition of PARP-1 activity presents a promising strategy for mitigating cell death in Alzheimer’s disease (AD), Parkinson’s disease (PD), ischemic stroke, epilepsy, and other neurological disorders, several studies have indicated that targeted PARP-1 therapies in animal models of the nervous system may be associated with significant side effects that warrant careful consideration. For instance, mice with a deletion of PARP-1 exhibit impaired neurogenesis and schizophrenia-like symptoms (Hong et al., 2019). PARP-1 is a crucial protein involved in the repair of DNA damage within cells, and cell death is typically induced only when PARP-1 is excessively activated. Consequently, due to the dual role of PARP-1 in cellular processes, although PARP-1 inhibitors are currently employed in clinical settings for the treatment of tumors, the efficacy and safety of these drugs for neurological disorders require further investigation (Krietsch et al., 2013). Therefore, there is an urgent need to develop drugs that target proteins downstream of PARP-1 in the PARthanatos pathway.

To identify pharmacological agents capable of inhibiting PARthanatos and enhance their clinical applicability, we conducted a screening of currently approved clinical drugs using a PARthanatos cell model. Our findings indicate that medroxyprogesterone effectively inhibits PARthanatos. Mechanistic studies revealed that medroxyprogesterone directly binds to extracellular signal-regulated kinase (ERK) and stabilizes the active phosphorylated ERK, thereby inhibiting apoptosis-inducing factor (AIF) translocation. Furthermore, the neuroprotective efficacy of medroxyprogesterone was validated in a mouse CIS model.

## 2 Results

### 2.1 Screening of the approved drug library to find the inhibitors of MNNG

**FIGURE 1:**
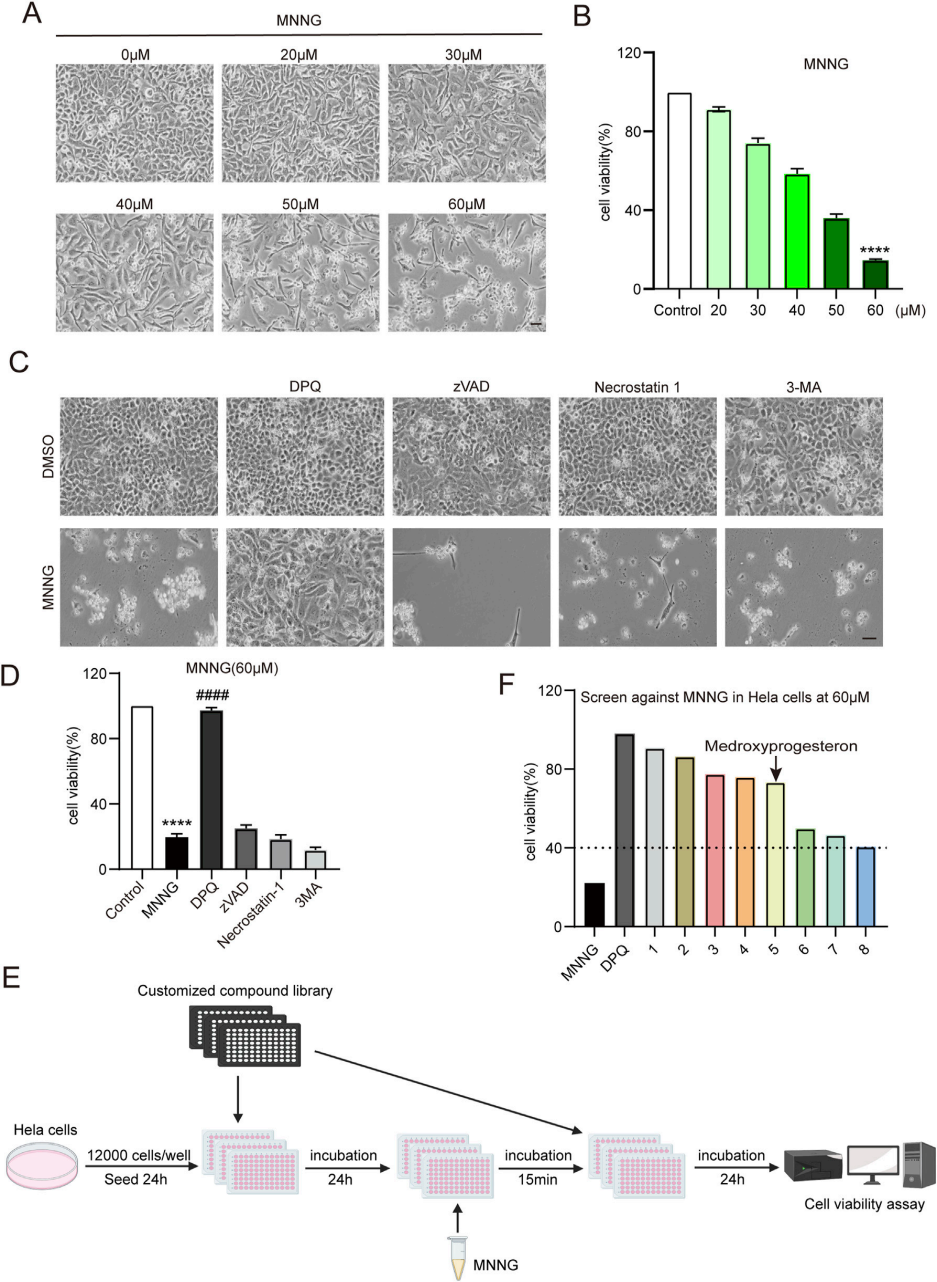
Compound library screening experiments. **(A, B)** HeLa cells were treated with different concentrations of MNNG (0–60 μM) for 15 min, then removed, and continued to be cultured in a complete medium for 24 h. **(A)** Representative cell images; scale bar, 25 μm. **(B)** Quantification of cell viability. **(C, D)** HeLa cells pretreated with cell death inhibitors DPQ (30 μM), z-VAD (20 μM), necrostatin-1 (20 μM), or 3-MA (2 mM) for 24 h were treated with DMSO or MNNG (60 μM, 15 min). Representative cell images of HeLa cells after being cultured with cell death inhibitors for 24 h. Scale bar, 25 μm **(C)**. The effect of cell death inhibitors on the cell viability rate in MNNG-induced HeLa cells was determined **(D)**. The data are presented as the mean ± SD (n = 3) (****P < 0.0001 vs. Control, ^####^P < 0.0001 vs. MNNG). **(E)** Flowchart of screening drugs that can reduce the toxic effect of MNNG on HeLa cells in the compound library. **(F)** Screening results showed eight compounds with a normalized viability rate of at least 40% after MNNG treatment in HeLa cells.

Initially, we exposed HeLa cells to increasing concentrations of MNNG (20–60 μM) for 15 min, followed by a 24-h recovery period in a growth medium. Our observations indicated a decrease in the number of cells adhering to the bottom of the plate as the concentration of MNNG increased (Figure 1A). The CCK-8 assay results (Figure 1B) demonstrated that at an MNNG concentration of 60 μM, cell viability reduced to approximately 14%, suggesting substantial cellular damage. Consequently, a concentration of 60 μM was selected for the MNNG treatment in subsequent experiments.

To elucidate the cell death pathway following MNNG induction, we investigated the impact of various cell death inhibitors on MNNG-induced HeLa cells. The findings (Figures 1C, D) revealed that only the PARP inhibitor DPQ (30 μM) was capable of completely inhibiting cell death triggered by MNNG (60 μM, 15 min). In contrast, inhibitors targeting apoptosis (z-VAD, 20 μM), necroptosis (necrostatin-1, 20 μM), and autophagy (3-methyladenine, 2 mM) were ineffective in preventing cell death. These results suggest that MNNG induces cell death through the mechanism of PARthanatos.

To identify pharmacological agents that can confer protection against the effects of MNNG, we conducted a screening of the approved drug library (Supplementary Table 1) (Yang et al., 2024) at a concentration of 60 μM (Figure 1E). In the first round of screening, we identified eight compounds with a normalized viability rate of at least 40% after MNNG treatment in HeLa cells (Figure 1F).

### 2.2 Medroxyprogesterone protects against the MNNG effect

**FIGURE 2:**
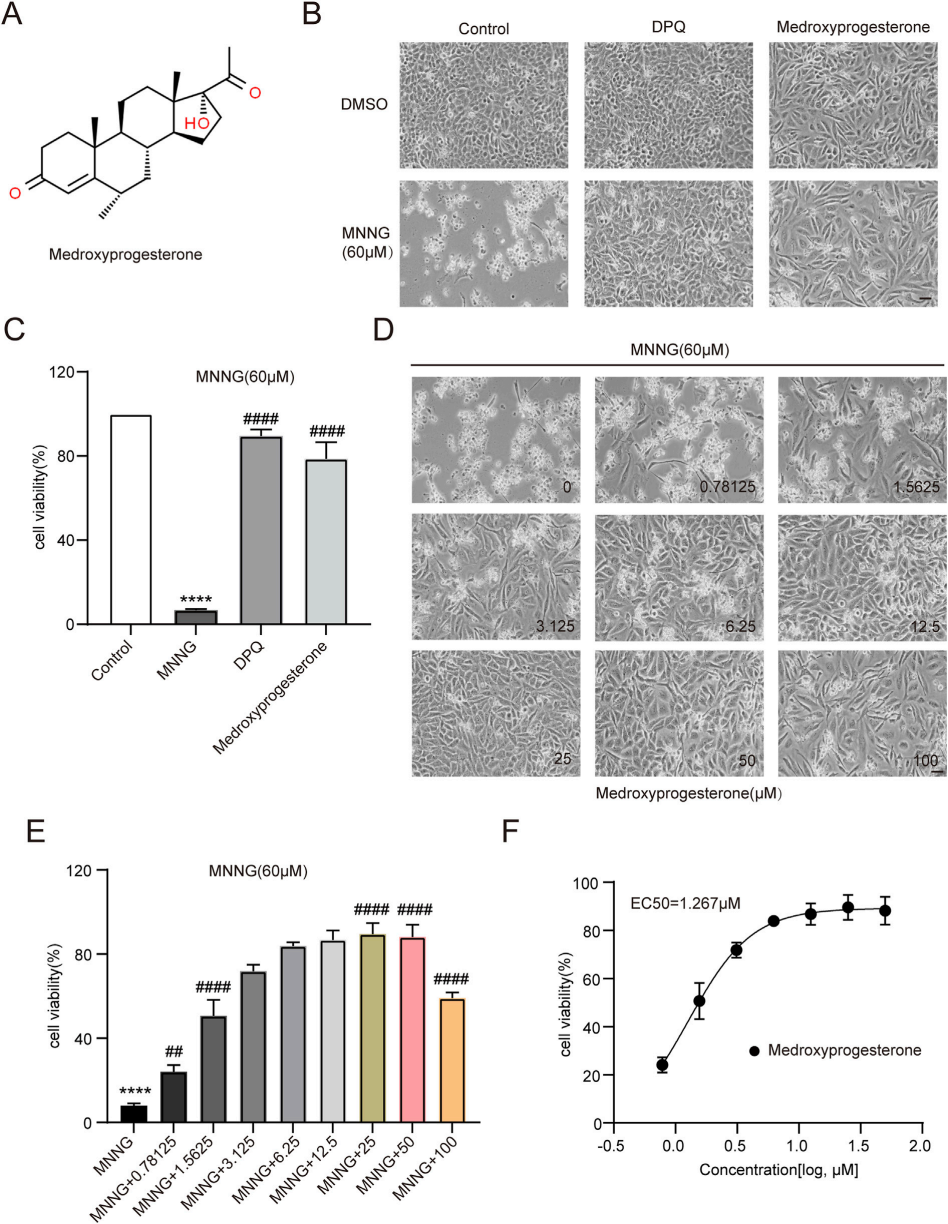
Effect of medroxyprogesterone on MNNG-induced cell death. **(A)** Chemical structures of medroxyprogesterone. **(B, C)** HeLa cells were exposed to MNNG (60 μM, 15 min) after 24 h of treatment with medroxyprogesterone. **(B)** Representative cell images after 24 h of MNNG treatment. **(C)** Cell viability after 24 h of MNNG treatment was measured using the CCK-8 assay. **(D, F)** HeLa cells pretreated with gradient concentration medroxyprogesterone (0–100 μM) for 24 h were exposed to MNNG (60 μM, 15 min). **(D)** Representative cell images of continuous treatment with medroxyprogesterone for 24 h. Scale bar, 25 μm. **(E)** The effect of different concentrations of medroxyprogesterone on HeLa cell viability after 24 h of MNNG treatment was determined by CCK-8 detection. **(F)** Concentration–cell viability data in **(E)** were curve-fitted to determine the EC_50_ value of medroxyprogesterone. EC_50_ = 1.267 μM (GraphPad Prism 10.1.1). The data are presented as the mean ± SD (n = 3) (****P < 0.0001 vs. Control, ^##^P < 0.01, ^####^P < 0.0001 vs. MNNG.).

After confirmation, 3F06 was identified as having a significant protective effect against MNNG toxicity. 3F06 was identified as medroxyprogesterone, a synthetic derivative of progesterone. The structure of medroxyprogesterone is shown in Figure 2A.

HeLa cells pretreated with medroxyprogesterone exhibited an approximately 80% increase in cell viability compared to the normalized value for the effect of MNNG alone, thereby significantly mitigating the cytotoxic impact of MNNG on the cells (Figures 2B, C). To determine the optimal concentration of medroxyprogesterone, we evaluated concentrations ranging from 0 to 100 μM, assessing cell survival and measuring cell viability. The protective effect of medroxyprogesterone was found to be concentration-dependent, with maximum efficacy observed at 25 μM (Figures 2D, E) and an EC_50_ value calculated to be 1.267 μM (Figure 2F).

### 2.3 Medroxyprogesterone prevents DNA fragmentation induced by MNNG

**FIGURE 3:**
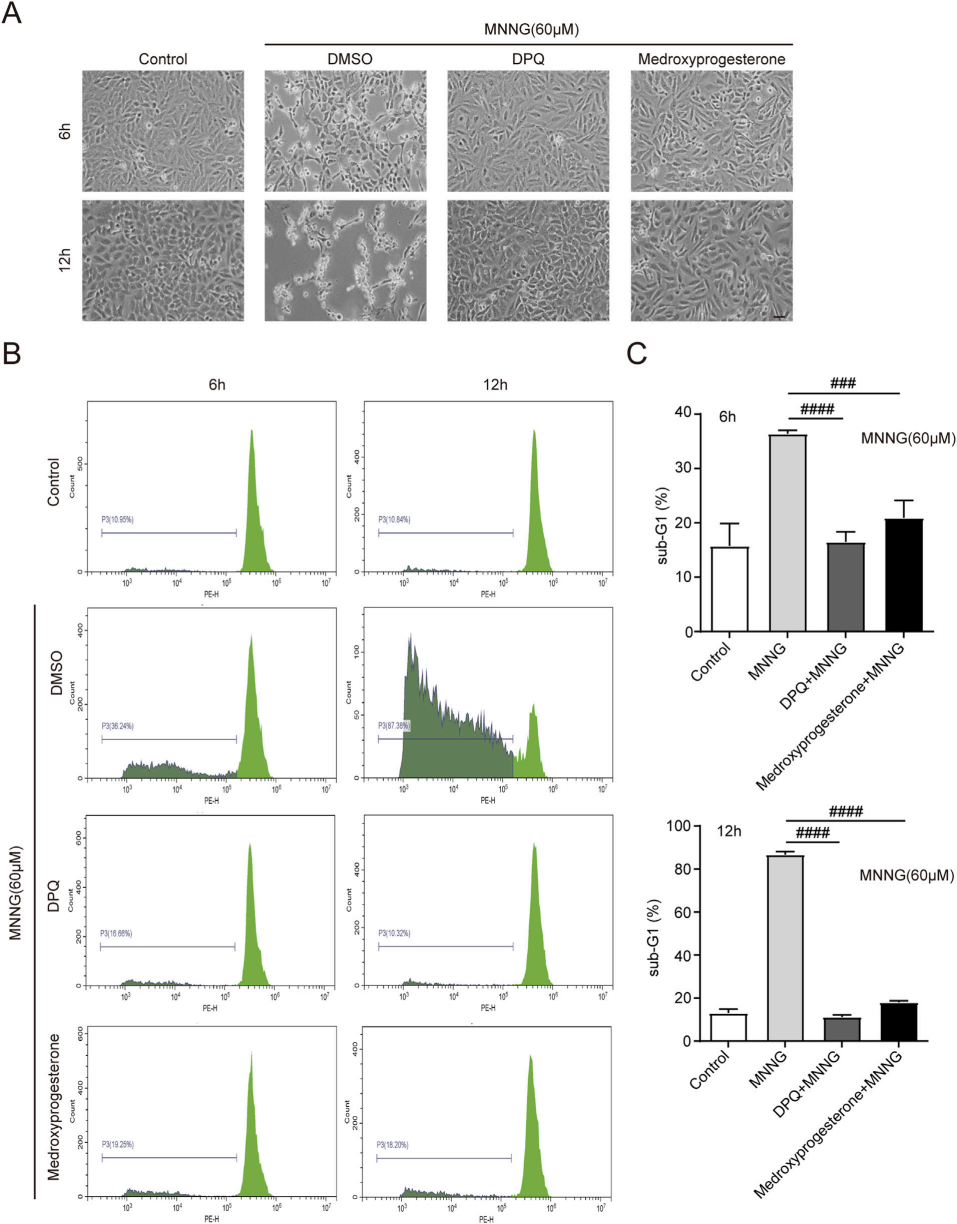
HeLa cells were treated with MNNG (60 μM, 15 min) in the presence or absence of pretreatment of medroxyprogesterone (25 μM) for 24 h. **(A)** Representative cell death images of HeLa cells after 6 h or 12 h of MNNG (60 μM, 15 min) treatment. Scale bar, 25 μm. **(B)** After treatment with MNNG (60 μM, 15 min) in the presence or absence of pretreatment medroxyprogesterone (25 μM) for 6 h or 12 h, HeLa cells were subjected to sub-G1 analysis by flow cytometry. **(C)** Columnar statistical chart of flow cytometry. The data are presented as the mean ± SD (n = 3) (^###^P < 0.001, ^####^P < 0.0001 vs. MNNG).

After the occurrence of PARthanatos in cells, AIF is released from mitochondria and subsequently carries MIF nuclease into the nucleus (Wang et al., 2016). After the action of endonucleases such as MIF, the chromosomes are decomposed into many oligonucleosomes within the nucleus. Due to their low molecular weight, these molecules can traverse both the nuclear and cell membranes, following detergent treatment of the cells. Consequently, a noticeable reduction in DNA content is observed, accompanied by the emergence of a sub-G1 peak in the histogram of nuclear DNA content, as determined by PI staining (Crowley et al., 2016). Cells were harvested for PI staining at 6 and 12 h post-MNNG treatment (Figure 3A). Our findings indicate that medroxyprogesterone treatment significantly diminishes the sub-G1 peak (Figures 3B, C), suggesting that a reduction in chromosomal DNA degradation in HeLa cells, following MNNG exposure, can be significantly inhibited by medroxyprogesterone.

### 2.4 Screening of five medroxyprogesterone analogs

**FIGURE 4:**
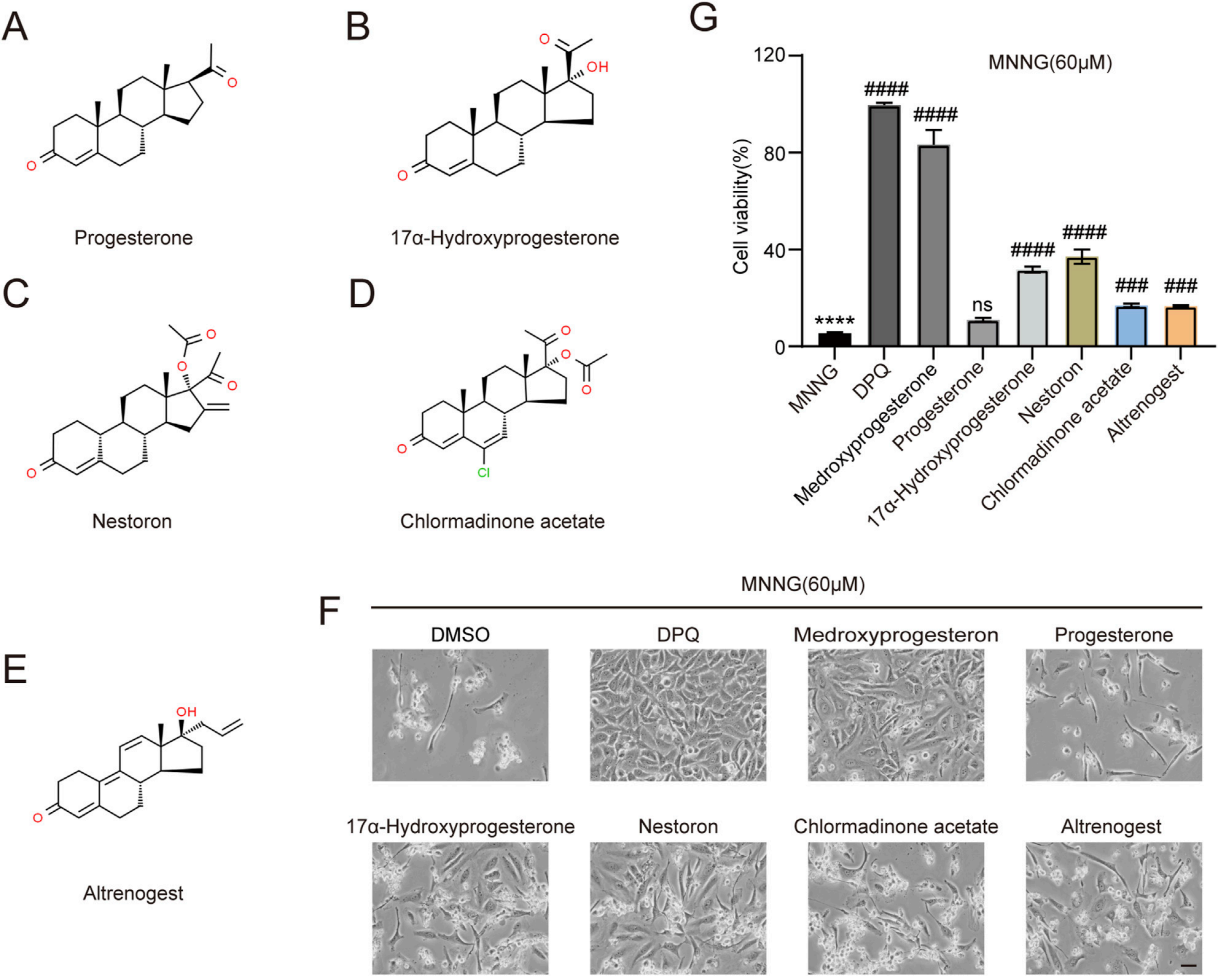
Screening of five medroxyprogesterone analogs. Chemical structures of progesterone **(A)**, 17α-hydroxyprogesterone **(B)**, nestoron **(C)**, chlormadinone acetate **(D)**, and altrenogest **(E)**. All structures are from ChemSpider. **(G, F)** HeLa cells were treated with DPQ (30 μM), medroxyprogesterone (25 μM), progesterone (10 μM), 17α-hydroxyprogesterone (50 μM), nestoron (50 μM), chlormadinone acetate (10 μM), and altrenogest (50 μM), respectively, for 24 h and then exposed to MNNG (60 μM, 15 min). **(F)** Representative images after 24 h of MNNG (60 μM, 15 min) treatment. Scale bar, 25 μm. **(G)** The effect of five medroxyprogesterone analogs on the cell viability rate in HeLa cells after 24 h of MNNG (60 μM, 15 min) treatment was determined by CCK-8 detection. The data are presented as the mean ± SD (n = 3) (****P < 0.0001 vs. Control, ^###^P < 0.001, ^####^P < 0.0001 vs. MNNG.).

We investigated whether structurally analogous compounds to medroxyprogesterone could similarly confer protection against the effects of MNNG in HeLa cells. Five compounds were selected for this study, namely, progesterone, 17α-hydroxyprogesterone, nestoron, chlormadinone acetate, and altrenogest (Figures 4A–E). The findings are presented in Figures 4F, G. The parent compound, progesterone, does not influence MNNG toxicity. Although the four progesterone derivatives exhibit inhibitory effects on MNNG toxicity, their efficacy is inferior to that of medroxyprogesterone, which incorporates methyl and hydroxyl groups based on the progesterone structure. Given that progesterone, the primary ligand of the progesterone receptor *in vivo*, is unable to inhibit the occurrence of PARthanatos in cells, it is proposed that medroxyprogesterone does not prevent PARthanatos through its function as a progesterone receptor ligand. Consequently, we aim to further elucidate the specific mechanisms by which medroxyprogesterone inhibits PARthanatos. The induction of PARthanatos by MNNG involves several key steps, including 1) the activation of PARP1 and a subsequent increase in protein PARylation; 2) alterations in mitochondrial membrane potential and permeability; 3) the cleavage of AIF by calpain or its modification by PAR, leading to its translocation from the mitochondria to the cytoplasm; 4) the subsequent association of AIF with MIF in the cytoplasm and their joint translocation to the nucleus; and 5) the cleavage of double-stranded DNA by endonucleases such as MIF, culminating in chromosomal degradation and cell death. By investigating these steps, we aim to identify the specific target protein of medroxyprogesterone acetate.

### 2.5 Medroxyprogesterone prevents MNNG-induced PARthanatos by blocking AIF translocation

**FIGURE 5:**
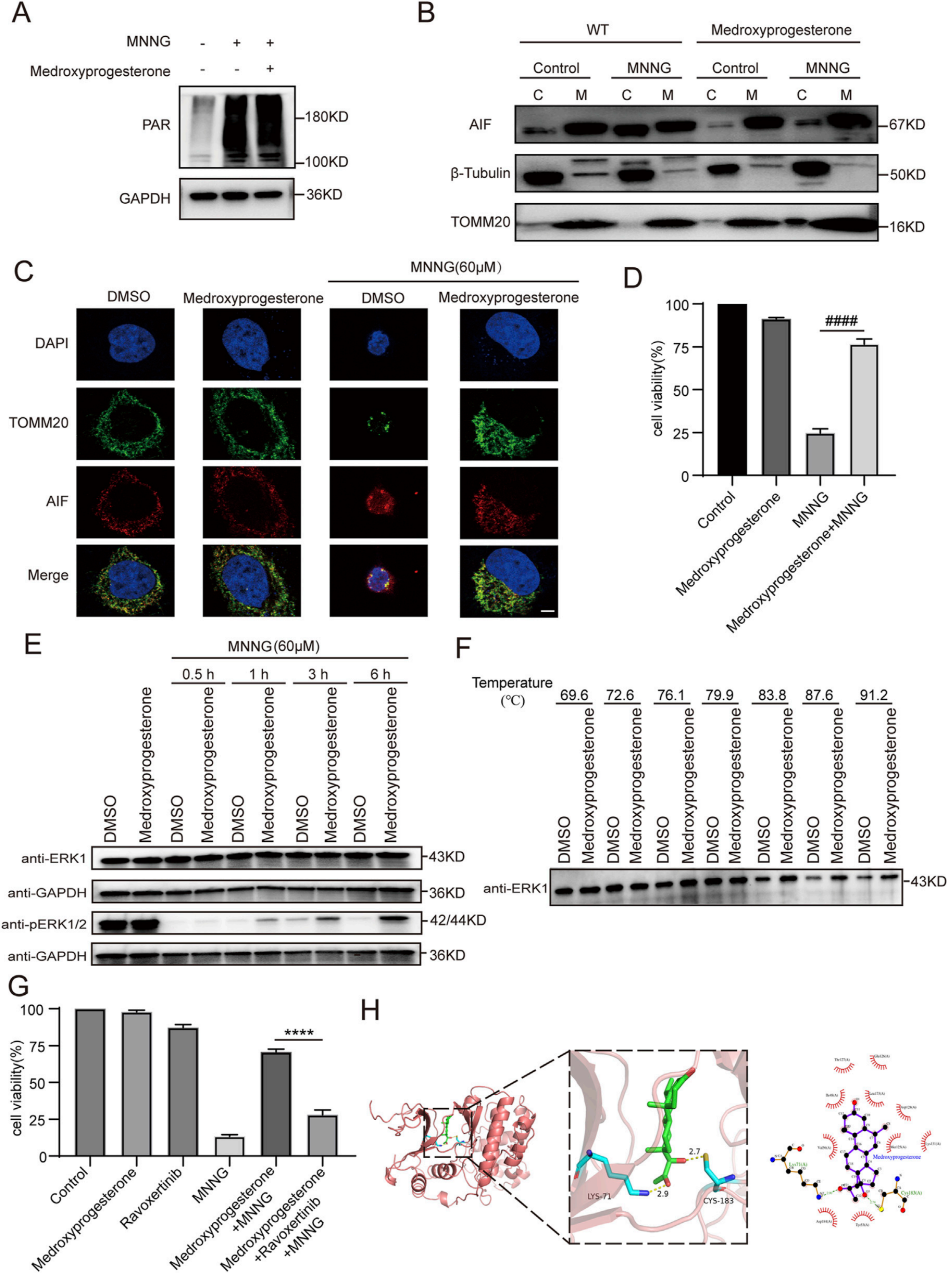
Effect of medroxyprogesterone on MNNG-induced PARthanatos. HeLa cells were treated with MNNG (60 μM, 15 min) in the presence or absence of pretreatment of medroxyprogesterone (25 μM) for 24 h. **(A)** Immunoblot analysis of the levels of PAR in HeLa cells after 30 min of MNNG (60 μM, 15 min) treatment. **(B)** HeLa cells after 6 h of MNNG (60 μM, 15 min) treatment were subjected to mitochondria isolation, and immunoblotting was performed with mitochondria (M) and cytoplasm **(C)** fractions. TOMM20 and β-tubulin were used as mitochondria and cytoplasm marker proteins, respectively. **(C)** Representative confocal images of the effect of medroxyprogesterone on MNNG-induced AIF translocation after 6 h of MNNG (60 μM, 15 min) treatment. Scale bar, 5 μm. **(D)** Cell viability after 6 h of MNNG (60 μM, 15 min) treatment was measured using the CCK-8 assay. The data are presented as the mean ± SD (n = 3) (^####^P < 0.0001 vs MNNG). **(E)** Immunoblot analysis of the levels of ERK1 and phosphorylation of ERK1/2 in HeLa cells after 0.5 h, 1 h, 3 h, and 6 h of MNNG (60 μM, 15 min) treatment (n = 3). **(F)** The thermal stability of ERK in HeLa cell lysate collected after 24 h pretreatment with medroxyprogesterone (50 μM) or DMSO was detected by Western blot. **(G)** HeLa cells were pretreated with medroxyprogesterone (25 μM) alone or medroxyprogesterone (25 μM) combined with ravoxertinib (1 μM) for 24 h. Cell activity was measured using CCK8 at 6 h after with to or without exposure to MNNG (60 μM, 15 min). The data are presented as the mean ± SD (n = 3) (****P < 0.0001 vs. medroxyprogesterone + MNNG). **(H)** Representative structure of medroxyprogesterone and ERK1 after molecular docking (left). Relative interactions between medroxyprogesterone and two amino acids of ERK1 generated by LigPlot (right).

First, we detected changes in the expression levels of key proteins in PARthanatos induced by MNNG in HeLa cells. The results indicated no change in the level of PAR modification (Figure 5A), indicating that medroxyprogesterone did not affect the activation of PARP-1 during PARthanatos. Medroxyprogesterone did not affect the expression of AIF and MIF proteins involved in the PARthanatos process either (Supplementary Figure 1).

We subsequently delved deeper into whether medroxyprogesterone could thwart PARthanatos by influencing AIF translocation. Our findings revealed that following medroxyprogesterone administration, AIF was unable to migrate from the mitochondria to the cytoplasm, effectively halting the progression of MNNG-induced PARthanatos (Figures 5B, C). Concurrently, CCK8 assays on HeLa cells indicated that medroxyprogesterone markedly enhanced cell viability by inhibiting MNNG-induced AIF translocation (Figure 5D). Utilizing SwissTARGET, we forecasted potential protein targets of medroxyprogesterone (Supplementary Table 2) and identified ERK as the prime candidate, a protein previously linked to MNNG-induced AIF release (Ethier et al., 2007). Immunoblot analyses conducted on HeLa cells treated with MNNG (60 μM, 15 min) for durations of 0.5 h, 1 h, 3 h, and 6 h revealed that medroxyprogesterone substantially mitigated the decrease in ERK phosphorylation triggered by MNNG (Figure 5E). Moreover, thermal stability assessments of ERK in HeLa cell lysates, pre-incubated for 24 h with either medroxyprogesterone or DMSO, corroborated medroxyprogesterone’s capacity to bind with ERK and shield it from degradation (Figure 5F). Intriguingly, ravoxertinib, an ERK inhibitor, was found to significantly counteract the protective effects of medroxyprogesterone (Figure 5G; Supplementary Figure 2). Molecular docking studies between medroxyprogesterone and ERK1 (Figure 5H) yielded a favorable binding energy of −9.5 kcal/mol for their optimal docking configuration. In the active conformation, a conserved lysine residue from the β3-strand (ERK1/2 K71/54) forms a stabilizing salt bridge with a conserved glutamate from the αC-helix (ERK1/2 E^88^/71); the absence of this salt bridge renders ERK1/2 inactive (Roskoski, 2012). Notably, the hydrogen bond formed between medroxyprogesterone and lysine plays a pivotal role in maintaining the integrity of this salt bridge.

### 2.6 Medroxyprogesterone preconditioning significantly protects neurons from cerebral ischemia in mice

The middle cerebral arterial occlusion (MCAO) model is the most widely used experimental paradigm for inducing cerebral ischemic stroke as it allows for transient occlusion by blocking the blood flow in the middle cerebral artery. However, the conventional MCAO model requires a high level of technical expertise from the operator, which can challenge the consistency of experimental reproducibility. Additionally, this model primarily mimics clinical scenarios where patients receive timely thrombolytic or thrombectomy interventions. In reality, a significant proportion of patients with cerebral ischemia do not receive such timely interventions and, thus, remain in a prolonged ischemic state. Therefore, we adopted a method reported in the literature (Jia et al., 2017) to induce focal ischemia by accurately controlling the infarct site and occlusion time through the aggregation of magnetic particles (MPs) mediated by micro-magnets in microvessels.

**FIGURE 6:**
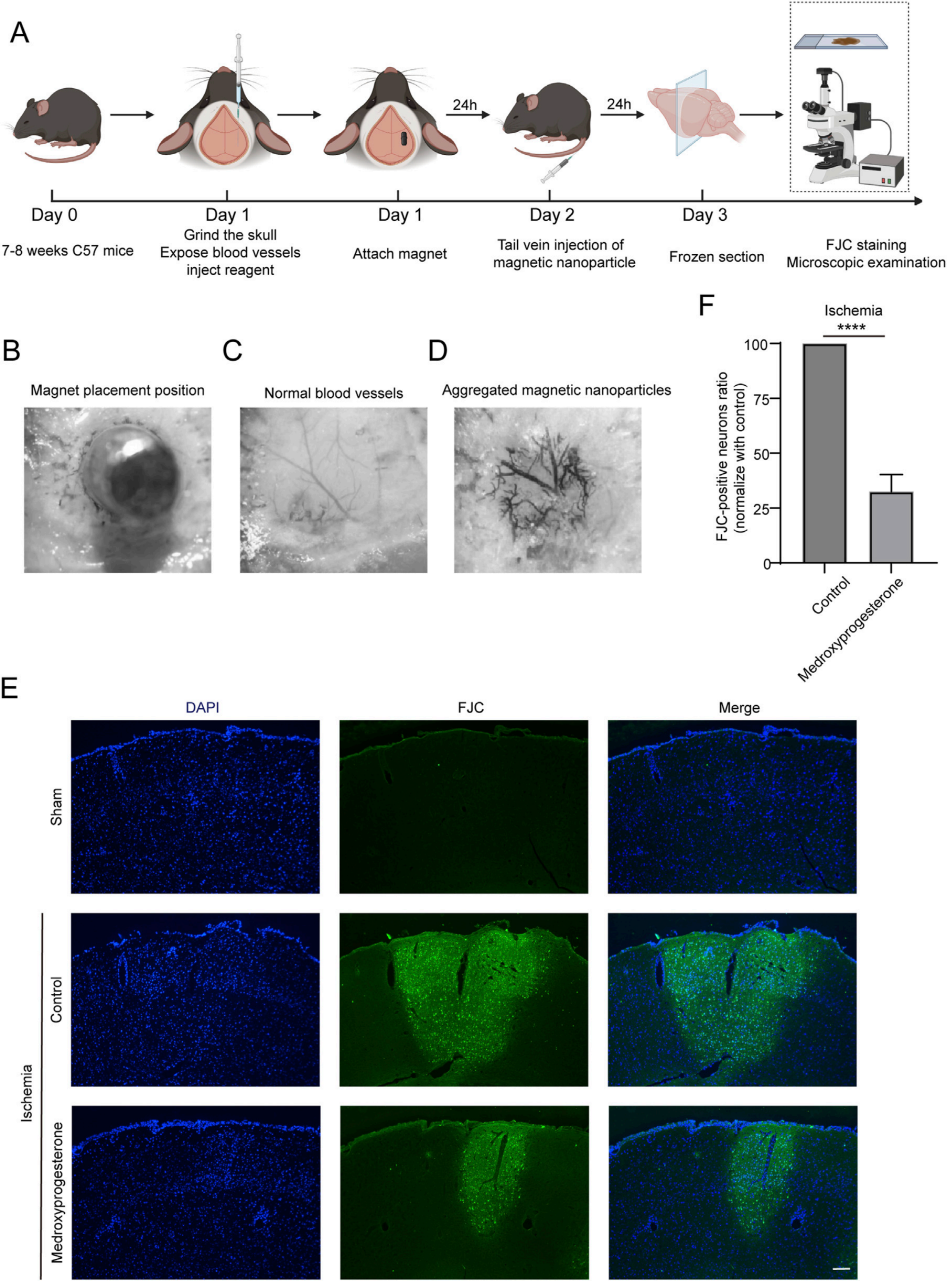
Pre-treatment with medroxyprogesterone inhibits neuronal degeneration after cerebral ischemia. **(A)** Schematic diagram of the experimental process of cerebral ischemia in mice. **(B–D)** Neodymium magnet column induces magnetic nanoparticle to aggregate in the cerebral cortex of mice. **(E)** At 24 h after MP-mediated occlusion, brain sections of the sham group, pretreated with normal saline or medroxyprogesterone (25 μM) for 24 h, were, respectively, labeled with FJC (blue fluorescence indicates the nucleus, and green fluorescence indicates the FJC-positive degenerative neurons; scale bar, 200 μm). **(F)** The FJC-positive neurons were statistically analyzed. The data are presented as the mean ± SD (n = 5) (****P < 0.0001 vs. control.).

The experimental procedure for inducing cerebral ischemia in mice is illustrated in Figure 6A. Initially, the skull of C57 mice is carefully ground to expose the blood vessels. Subsequently, a site adjacent to the main trunk of the posterior cerebral artery is identified, where 1 μL of either physiological saline or medroxyprogesterone (25 μM) is injected. A magnet is then positioned above the injection site, as depicted in Figure 6B. After 24 h, normal blood vessels (Figure 6C) become occluded due to the aggregation of MPs, following tail vein injection (Figure 6D). Following 24 h of ischemia, we assessed the presence of PARthanatos-related proteins in the ischemic regions. The results indicated an increase in PAR following ischemic treatment. In comparison to the control group, pretreatment with medroxyprogesterone did not significantly alter the expression levels of PAR, AIF, and MIF (Supplementary Figures 3A–C); however, it effectively inhibited the translocation of AIF (Supplementary Figure 3D). FJC staining (Figures 6E, F) revealed positive staining in the ischemic mouse model, indicating cortical neuron damage. Notably, the pre-administration of medroxyprogesterone significantly mitigated neuronal death in the ischemic injury region.

**FIGURE 7:**
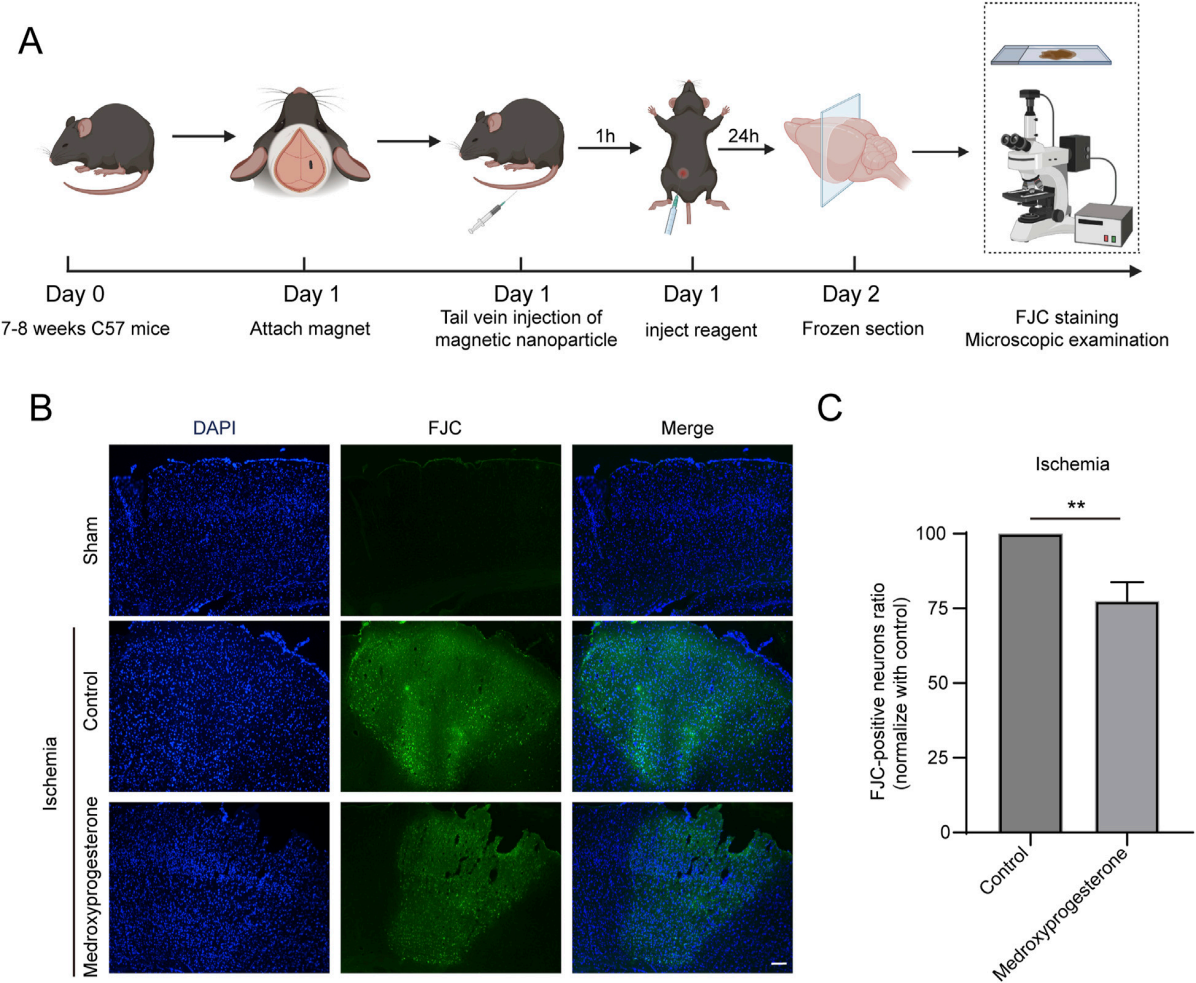
Post-treatment with medroxyprogesterone inhibits neuronal degeneration after cerebral ischemia. **(A)** Schematic diagram of the experimental process of medroxyprogesterone (5 mg/kg/day) intervention after cerebral ischemia in mice. **(B)** At 24 h, after MP-mediated occlusion, brain sections of the sham group, injected with normal saline or medroxyprogesterone (5 mg/kg/day) 1 h after ischemia were, respectively, labeled with FJC (blue fluorescence indicates the nucleus, and green fluorescence indicates the FJC-positive degenerative neurons; scale bar, 200 μm). **(C)** The FJC-positive neurons were statistically analyzed. The data are presented as the mean ± SD (n = 3) (**P < 0.01 vs. control.).

We also sought to further understand whether medroxyprogesterone could effectively protect neurons after cerebral ischemia. To this end, we administered medroxyprogesterone intraperitoneally 1 h after cerebral ischemia. Our findings indicate that this post-ischemic treatment still significantly reduced neuronal death in the ischemic region, with a reduction of approximately 20%–30% (Figures 7A–C), compared to a 60%–70% reduction with medroxyprogesterone pre-treatment.

## 3 Discussion

In 2007, Ted Dawson and Valina Dawson of the Johns Hopkins University School of Medicine discovered a new form of regulatory cell death PARthanatos, named after PAR (the living product of PARP enzyme) and Thanatos (the Greek god of death), also known as PARP-1 dependent cell death. The use of caspase inhibitors such as z-VAD-fmk cannot block the occurrence of PARthanatos (Yu et al., 2002; Wang et al., 2019). Cerebral ischemic injury results in the release of substantial amounts of the excitatory neurotransmitter glutamate, which subsequently activates N-methyl-D-aspartate (NMDA) receptors. This activation leads to an influx of calcium ions and the production of nitric oxide (NO) and ROS. NO rapidly reacts with superoxide to form the highly unstable oxidant peroxynitrite (ONOO-), which induces chromosomal DNA strand breaks, thereby triggering the activation of PARP-1 (Liu et al., 2022).

PARP-1 exhibits dual functions. Primarily, it serves a protective role by recruiting DNA repair-related proteins through PAR modification, thereby playing a crucial role in DNA base excision repair, single-strand break repair, and non-homologous end joining. This activity prevents the accumulation of potentially lethal DNA double-strand breaks and promotes cell survival (Krietsch et al., 2013). Additionally, PARP-1 is involved in promoting cell death, contributing to overall organismal health by facilitating the translocation of AIF from the mitochondria to the nucleus and subsequently activating nuclear processes, promoting DNA breakage, and causing cell death, thus eliminating defective cells that are considered irreparable.

Previous studies have tried to use PARP-1 inhibitors to protect damaged neuronal cells, which can maintain the survival of neuronal cells during the use of PARP-1 inhibitors (Jagtap and Szabo, 2005; Moroni et al., 2012). However, because the activity of DNA repair proteins recruited by PARP-1 is also inhibited, the damaged DNA cannot be repaired correctly, so once the PARP-1 inhibitors are removed, the cells that have accumulated significant DNA damage will continue to develop PARthanatos (Hong et al., 2019; Kim et al., 2021; Xu et al., 2023). This also leads to the limitation of the use of PARP-1 inhibitors in treating nervous system diseases.

Our study found that medroxyprogesterone does not inhibit PARP-1 activity, so medroxyprogesterone can protect neurons from death without affecting the repair of damaged DNA. Therefore, medroxyprogesterone cannot cause secondary neuronal death, even if it is removed after short-term application of medroxyprogesterone and blood flow reconstruction in cerebral ischemic areas.

We have discovered that medroxyprogesterone may suppress PARthanatos by inhibiting the release of AIF from mitochondria. The precise mechanism by which AIF is translocated from the mitochondria to the cytoplasm remains unclear. Some studies suggest that this process is dependent on CAPN1-mediated cleavage (Chelko et al., 2021); however, others propose that AIF translocation does not require CAPN1 cleavage but is instead regulated by PAR modifications (Wang et al., 2009; Wang et al., 2011). Despite these differing views, there is a lack of a comprehensive mechanism elucidating how PAR modifications facilitate AIF’s movement from the mitochondria to the cytoplasm. Further detailed research is necessary to establish how medroxyprogesterone influences AIF’s dissociation from the mitochondria and its subsequent cytoplasmic entry. Using SwissTARGET, we predicted potential protein targets and identified ERK as the most likely candidate. ERK is associated with MNNG-induced AIF release (Ethier et al., 2007). Our results indicate that medroxyprogesterone significantly inhibits the decrease in ERK phosphorylation induced by MNNG, and thermal shift assays confirm its binding with ERK. We speculate that medroxyprogesterone acts as an ERK agonist, stabilizing phosphorylated ERK, thereby inhibiting BAX aggregation and mitochondrial damage and, thus, preventing AIF release (Ethier et al., 2007; Wang et al., 2007; Cabon et al., 2012).

Because PARthanatos is the main form of neuronal death after cerebral ischemia (Koehler et al., 2021; Liu et al., 2022), we also clearly proved that medroxyprogesterone can significantly inhibit neuronal death at the injured site in the mouse cerebral ischemia model.

Medroxyprogesterone was used in estrogen replacement therapy in the early stages and for extended periods in postmenopausal women (Crandall et al., 2023). However, it was found that medroxyprogesterone had a weak thrombogenic effect, so the long-term use of medroxyprogesterone was considered to promote myocardial ischemia and cerebral ischemia (Lisabeth and Bushnell, 2012). However, our experimental findings suggest that the short-term application of medroxyprogesterone after cerebral ischemia will have a neuroprotective effect, which can prevent the death of neurons by inhibiting the occurrence of PARthanatos. Because medroxyprogesterone is a listed drug, its toxicity and side effects are small, and it is easy to use in clinical application. Medroxyprogesterone primarily exerts its effects by directly preventing neuronal death, distinguishing it from the neuroprotective agents butylphthalide and edaravone, currently utilized in clinical settings. Our research indicates that butylphthalide and edaravone do not inhibit cell death via PARthanatos (Supplementary Figure 4). Their mechanisms of action are likely associated with anti-inflammatory effects and the reduction in ROS production, which do not directly avert neuronal death in ischemic regions (Zhang et al., 2005; Zhang et al., 2022; Patel and Chauhan, 2024; Sun et al., 2024). Consequently, clinical observations reveal that neuronal cell death persists in stroke patients despite the administration of these drugs. This leads to the progressive worsening of motor dysfunction. However, medroxyprogesterone shows potential to directly inhibit neuronal death in ischemic regions. We hypothesize that the early administration of medroxyprogesterone in stroke patients may directly prevent neuronal death in ischemic areas and mitigate motor dysfunction. Additionally, it may extend the therapeutic window for thrombolytic agents without exacerbating neuronal death associated with reperfusion injury following thrombolysis. Given that medroxyprogesterone is a progestin, its application in female patients is generally unproblematic. However, in male patients post-stroke, it is crucial to evaluate the potential of medroxyprogesterone to induce breast hyperplasia and other adverse effects that may compromise male sexual characteristics. Consequently, there is a need to develop novel pharmacological agents to inhibit cell PARthanatos in male patients.

Our findings suggest that medroxyprogesterone could serve as an effective neuroprotective agent for individuals with cerebral ischemia, thereby facilitating patient recovery and reducing the social burden.

## 4 Materials and methods

### 4.1 Cell culture and treatment

HeLa cells were purchased from the Cell Bank, Chinese Academy of Sciences (Shanghai, China). All compounds were purchased from TargetMol (Shanghai, China). In a constant 37°C, 5% carbon dioxide incubator, HeLa cells were cultured in high-glucose DMEM (Vivacell, China) containing 10% FBS (Vivacell, China) and 1% penicillin–streptomycin (Biosharp, China). Treatment with 60 μm MNNG for 15 min was used to induce PARthanatos. When testing the effect of compounds on the toxicity of MNNG, HeLa cells were pretreated with the compound for 24 h.

### 4.2 Cell viability assay

After counting, cells were seeded into a 96-well plate with three duplicated wells at a density of 1.2 × 10^4^ cells per well. After treatment, 10 μL of Cell Counting Kit-8 reagent (CCK-8, Beyotime, China) was added to each well and incubated at 37°C for 2 h. After incubation, absorbance was measured at 450 nm. Cell viability was expressed by the proportion of the control group.

### 4.3 Flow cytometry

Flow cytometry was used to determine the cell cycle distribution and DNA fragmentation using a cell cycle kit with propidium iodide (PI) staining (BD Biosciences). HeLa cells were collected by centrifugation at 200 × g for 7 min at 4°C. Subsequently, the cells were washed twice with cold PBS, centrifuged at 500 × g for 5 min, stained with PI (50 mg/mL stock solution) containing 0.1% (w/v) Triton X-100, subjected to flow cytometry on a Beckman CytoFLEX Flow Cytometer, and analyzed using Beckman CytExpert software.

### 4.4 Molecular docking

Medroxyprogesterone’s potential protein targets were predicted on SwissTARGET (Daina et al., 2019) and further analyzed with bioinformatics (Xu et al., 2022). We used the X-ray crystal structure of ERK1 (Protein Data Bank code: 4QTB) and medroxyprogesterone (mol: 520-85-4). AutoDock Vina 1.1.2 software was used for molecular docking (Van Zundert et al., 2016), and LigPlot + software was used for chemical bond analysis (Laskowski and Swindells, 2011).

### 4.5 Cell thermal shift assay

Cells, collected after administration, were suspended in PBS containing protease and phosphatase inhibitors. The cell suspension was gradually heated and then transferred to liquid nitrogen to freeze and thaw three times to lyse the cells. The heated deformed protein was removed by centrifugation (13,000 g, 30 min), and the target protein was detected and quantified by Western blot.

### 4.6 Mitochondria isolation

After the digestion of the cells by pancreatic enzymes, the cells were collected by centrifugation at room temperature for 10 min at 100–200 g. The cells were precipitated with pre-cooled PBS suspension and centrifuged at 600 g, 4°C for 5 min, and the supernatant was discarded. The cells were suspended in the mitochondria isolation reagent (Beyotime, China) and incubated in ice for 15 min. The cells were transferred to a glass homogenizer and homogenized for 30 strokes, and then, the cell homogenate was centrifuged at 600 g and 4°C for 10 min. After absorbing the supernatant, the sample was centrifuged at 11,000 g and 4°C for 10 min, and the precipitate obtained was mitochondria. The supernatant was centrifuged at 12,000 g and 4°C for 10 min. After centrifugation, the supernatant was cytoplasm.

### 4.7 Immunofluorescence

Cells, after PBS cleaning, were fixed with 4% paraformaldehyde, permeabilized with 0.3% Triton X-100, and blocked with an immunostaining blocking solution (Beyotime, China). Cells were incubated with AIF (1:100) and TOMM20 (1:100) antibodies overnight and then incubated with a fluorescent secondary antibody. The nucleus was labeled using 4′,6-diamidino-2-phenylindole (DAPI). Fluorescence was observed using a confocal laser scanning microscope.

### 4.8 Western blotting

After collecting the cell pellet, RIPA lysis buffer (Beyotime, China) was added. The cells were lysed on ice, swirling every 5 min. After 30 min, the sample was centrifuged at 4°C and 12,000 rpm for 20 min to collect the supernatant. The protein concentrations were quantified using a BCA Protein Assay Kit (Beyotime, China). Protein samples containing the loading buffer were separated by SDS-PAGE gel electrophoresis and transferred to a PVDF membrane (MilliporeSigma, Germany). The membrane was placed in a rapid blocking buffer (Sharebio, China) for 15 min at room temperature. Then, the PVDF membrane was incubated overnight at 4°C with primary antibody: anti-PARP-1 (1:1,000, ABclonal, United States); anti-PAR (1:1,000, Bio-Techne, United States); anti-MIF(1:1,000, Cusabio, China); anti-AIF (1:1,000, Santa Cruz, United States); anti-β-Tubulin (1:1,000, Beyotime, China); anti-TOMM20 (1:1,000, Beyotime, China); anti-GAPDH (1:20,000, ABclonal, United States); anti-ERK1 (1:1,000, Beyotime, China); and anti-phospho-ERK1/ERK2 (1:1,000, Beyotime, China). After the incubation of the primary antibody, the membrane was washed with PBST and incubated with the HRP-conjugated antibody (1:5,000, ABclonal, United States) at room temperature for 2 h. After washing with PBST, the immunoreactive protein was observed with an enhanced chemiluminescence detection reagent using a gel imaging system (Bio-Rad, United States).

### 4.9 Mice

The C57BL/6J mice used in this study, aged 7–8 weeks, were obtained from Henan SKBEX Biotechnology Co., Ltd. (Henan, China). Animal experiments were conducted in accordance with the ARRIVE guidelines (PLoS Bio 8 (6), e1000412, 2010), the regulations on the management of experimental animals issued by the State Commission for Science and Technology, and the implementing rules on the management of medical experimental animals issued by the Ministry of Health. The Medical Ethics Committee of Wannan Medical College approved all animal experiments (WNMC-AWE-2024363).

### 4.10 Occlusion with micromagnets after thinned-skull preparation

Mice were anesthetized by inhaling 1.5% isoflurane. After removing scalp fur, we cut a 1.5-cm-long skin incision to expose the mouse skull. We thinned a 2-mm square area on the skull by removing the skull outer plate and plate barrier with a microdrill and attached a cylindrical micro-magnet of 1.5-mm diameter using Ergo 5210 glue. When the skull was thinned, the middle cerebral artery was clearly visible; a measure of 1 μL of medroxyprogesterone or normal saline was stereoscopically injected into the cerebral cortex of the perivascular area. After 24 h, PEG-2000-coated nanoparticles (Micromod, Germany) (180 nm, 10 mg/mL) were injected into the tail vein of mice.

### 4.11 Fluoro-Jade C staining

Mice were transcardially perfused with 0.9% NS, followed by 4% paraformaldehyde successively 24 h after occlusion. After the brain was taken, it was cut into 20-μm sections. The brain tissue was placed on a gelatin-coated slide and dried in an oven at 50°C–60°C for 30 min to 1 h. The slides were incubated for 5 min in a dyeing tank containing a 9:1 ratio of 80% ethanol to sodium hydroxide solution. After incubation, the slides were transferred to 70% ethanol and distilled water for 2 min each. A solution containing a 9:1 ratio of double steaming water to potassium permanganate was added to the slide and incubated for 10 min. After cleaning with distilled water, the slides were incubated for 10 min in a dyeing tank containing a 9:1 ratio of double steaming water to Fluoro-Jade C (Beyotime, China). The slides were rinsed with distilled water for 1 min and then dried in a constant temperature oven at 50°C–60°C for 5 min. The dried sections were mounted with DPX.

### 4.12 Statistical analysis

At least three independent experiments were performed for each analysis, and the results were presented as the mean ± standard deviation. All statistical analyses using one-way ANOVA were performed using GraphPad Prism 10.1.1.

## Data Availability

The original contributions presented in the study are included in the article/Supplementary Material; further inquiries can be directed to the corresponding authors.
