# Supplementary material for: Medroxyprogesterone promotes neuronal survival after cerebral ischemic stroke by inhibiting PARthanatos
Source: Front Pharmacol. 2025 Feb 13;16:1487436. doi: 10.3389/fphar.2025.1487436 (PMC11865058; doi:10.3389/fphar.2025.1487436)
Supplement: Supplementary file 1 [file DataSheet1.zip › Supplementary Material.docx]

Supplementary Material

# Supplementary Figure


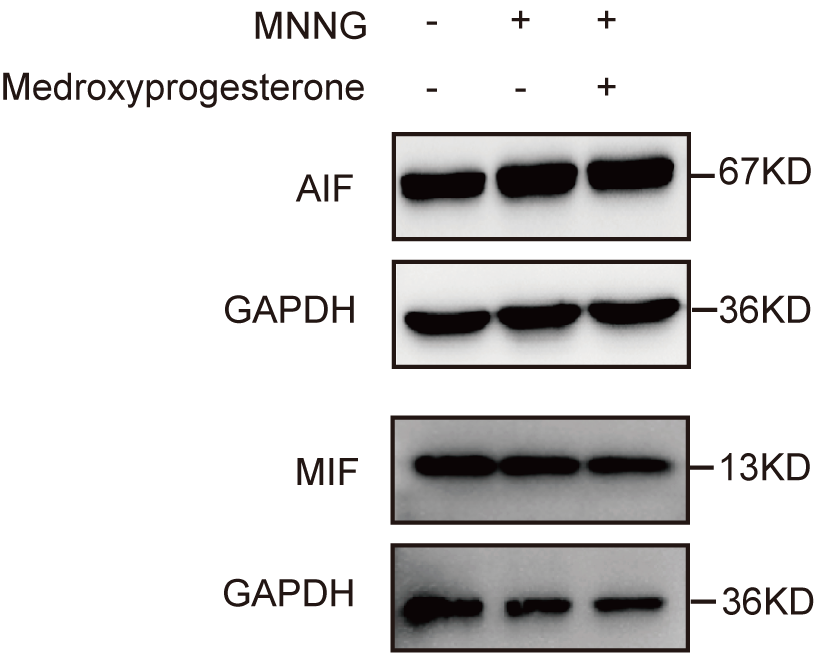


**Supplementary Figure 1.** Hela cells were treated with MNNG (60 μM, 15 min) in the presence or absence of pretreatment of medroxyprogesterone (25 μM) for 24 h. The expression of AIF and MIF in Hela cells was detected after 6 h of MNNG (60 μM, 15 min) treatment.


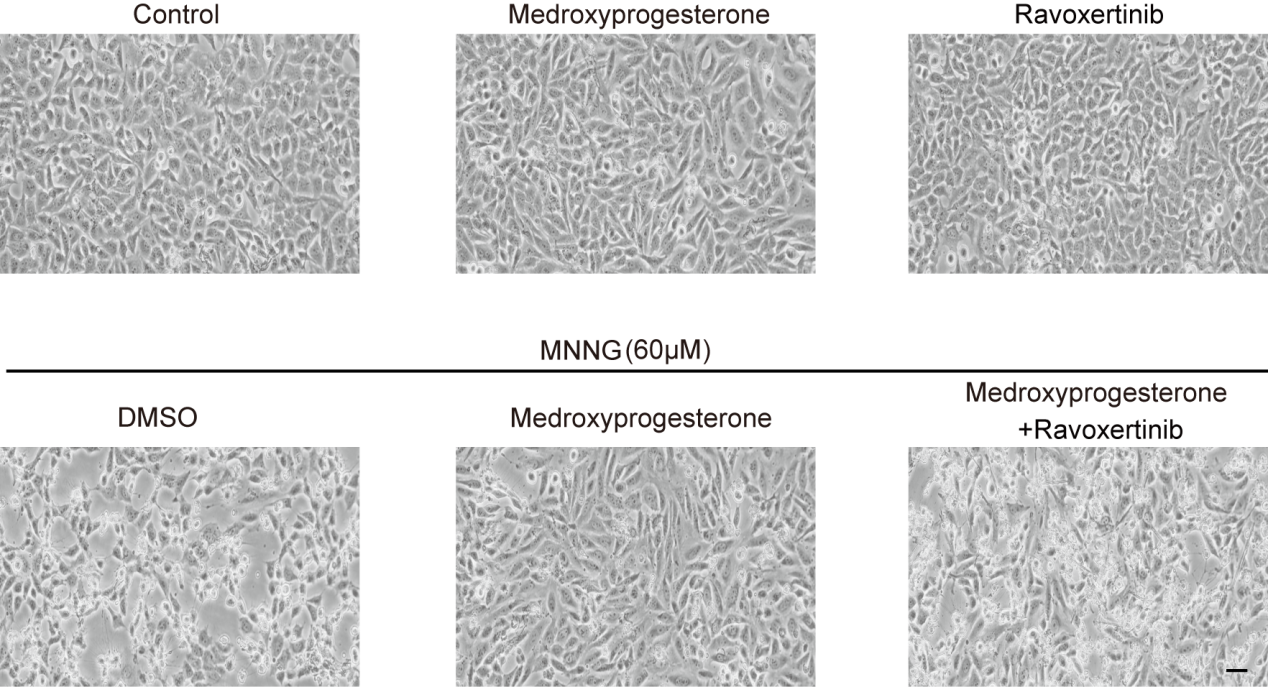


**Supplementary Figure 2.** Hela cells were pretreated with medroxyprogesterone (25 μM) alone or medroxyprogesterone (25 μM) combined with Ravoxertinib (1 μM) for 24 h. Representative images cells cultured with the drug for 6 h after MNNG (60 μM, 15 min) treatment, Scale bar, 50μm.


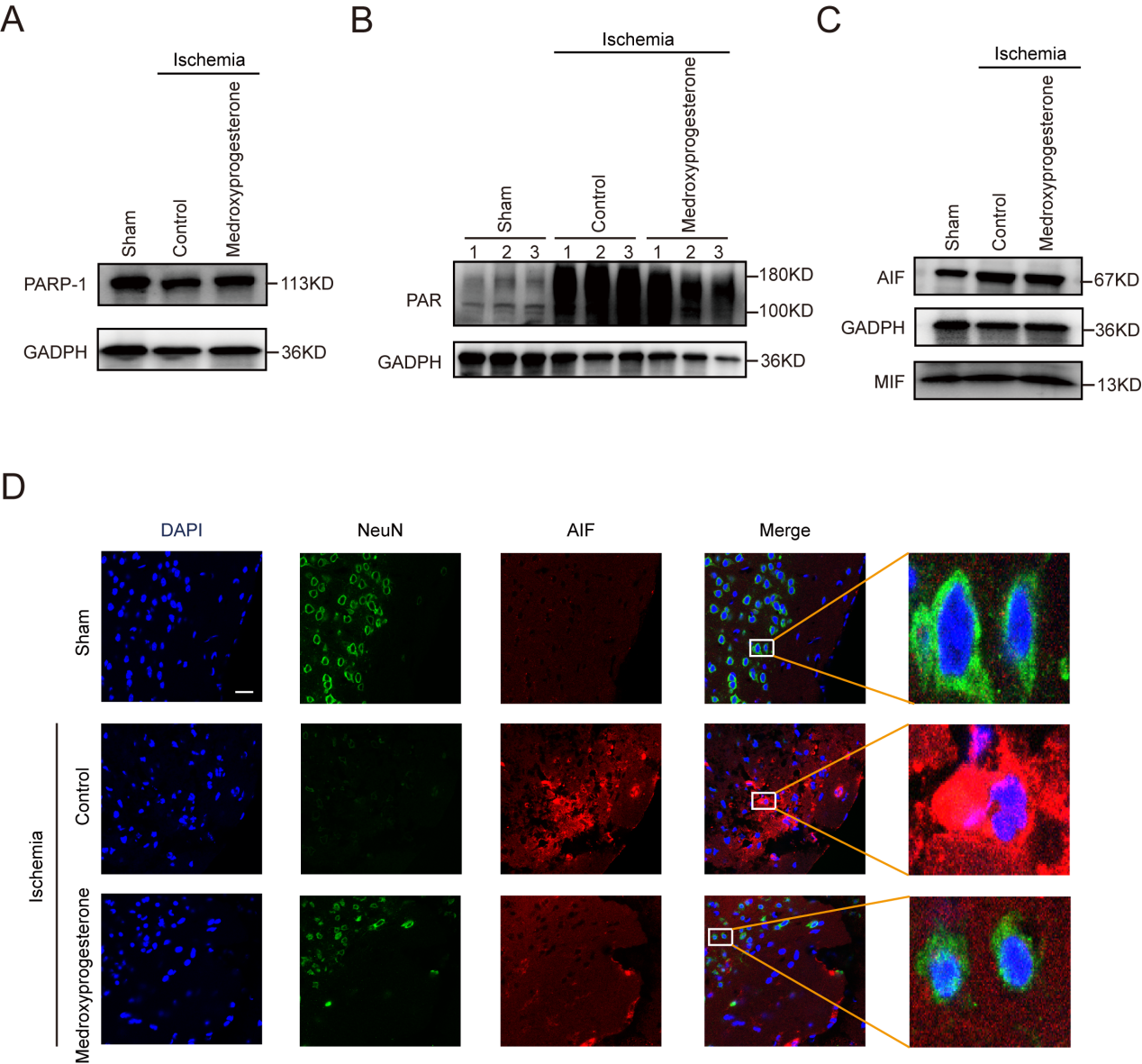


**Supplementary Figure 3.** After 24 h of normal saline or medroxyprogesterone (25 μM) pretreatment, magnetic nanoparticles were injected into the tail vein for ischemia. (A-C) At 24 h after MP-mediated occlusion, PARP-1 (A), PAR (B), AIF and MIF (C) expression. (D) At 24 h after MP-mediated occlusion, representative images of NeuN (green) and AIF (red) immunofluorescence with DAPI (blue) staining, Scale bar, 20 μm.


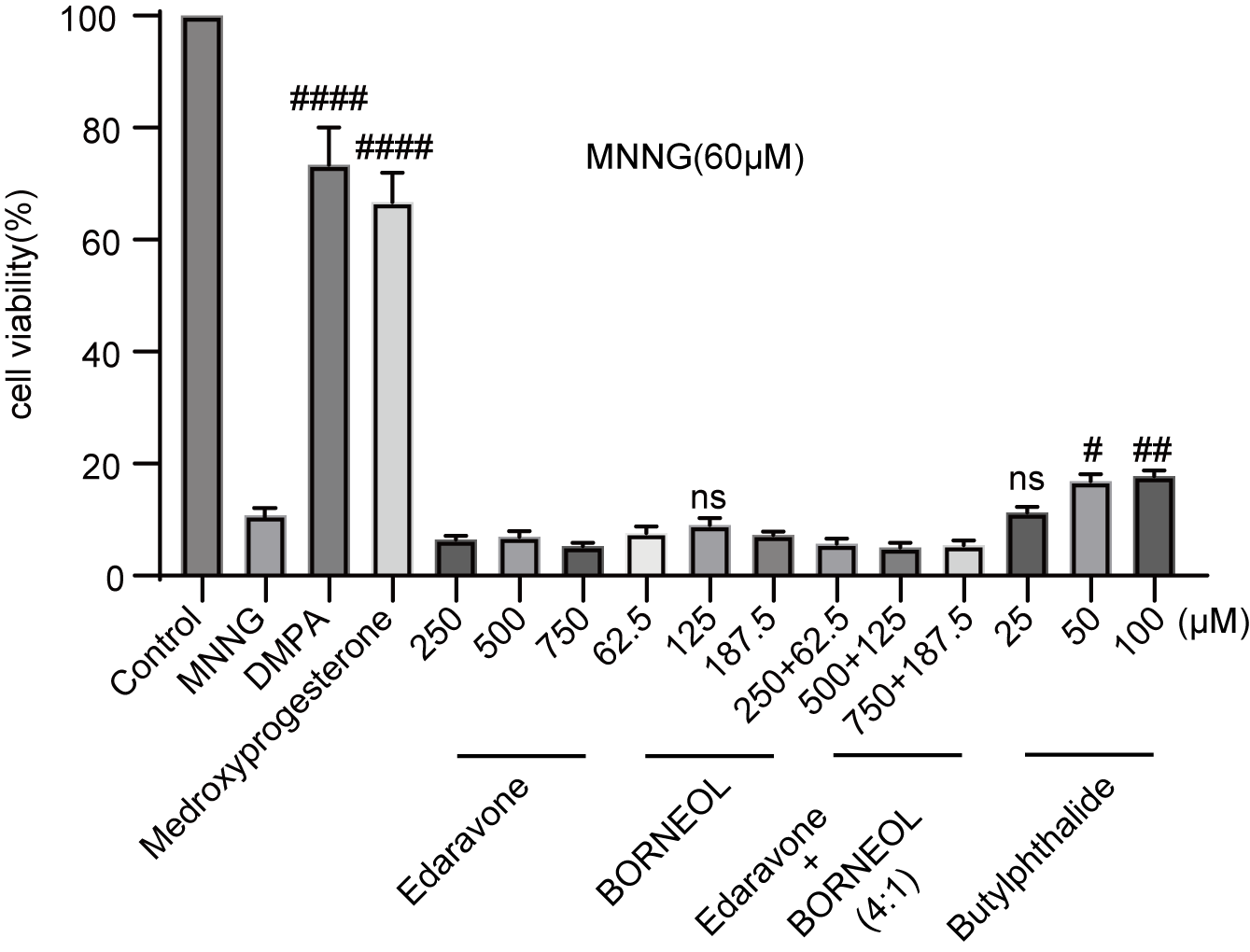


**Supplementary Figure 4.** DMPA (25 μM), medroxyprogesterone (25 μM), Edaravone, BORNEOL, Edaravone combined with BORNEOL (4:1) and Butylphthalide pretreated Hela cells for 24 h, respectively. The drug continues to treat the cells for 24 h after MNNG treatment (60 μM, 15 min). The cell viability of each group was detected by CCK8 assay. (The data are presented as the mean ± SD (n=3) (^#^P < 0.05, ^##^P < 0.01, ^####^P < 0.0001 vs. MNNG.)
