# Supplementary material for: Medroxyprogesterone promotes neuronal survival after cerebral ischemic stroke by inhibiting PARthanatos
Source: Front Pharmacol. 2025 Feb 13;16:1487436. doi: 10.3389/fphar.2025.1487436 (PMC11865058; doi:10.3389/fphar.2025.1487436)
Supplement: Supplementary file 1 [file DataSheet1.zip › Table 1.docx]

| **Plate layout:PHD147295** | | | | | | | | | | | | | | | |
| --- | --- | --- | --- | --- | --- | --- | --- | --- | --- | --- | --- | --- | --- | --- | --- |
|  | 1 | | 2 | 3 | 4 | 5 | 6 | 7 | 8 | | 9 | | 10 | 11 | 12 |
| a | Empty | | T2205 | T7064 | T0685 | T0762 | T1301 | T1297 | T0461 | | T0443 | | T0433 | T0019 | Empty |
|  |  |  | Acefylline | Valproic Acid | Retinyl acetate | Trans-Anethole | Guaiacol | Eugenol | Berberine chloride | | Salicin | | Artesunate | Betaine chloride |  |
| b | Empty | | T0129 | T0130 | T0228 | T0278 | T0281 | T0295 | T0469 | | T0473 | | T0480 | T0492 | Empty |
|  |  |  | Sanguinarine chloride | Physostigmine Salicylate | Methyl-Hesperidin | Escin | Meclocycline sulfosalicylate salt | Digitoxin | Octopamine hydrochloride | | Arbutin | | Doxofylline | Dimethyl fumarate |  |
| c | Empty | | T0522 | T2847 | T2952 | T2865 | T2741 | T2979 | T0122 | | T2215 | | T2719 | T2900 | Empty |
|  |  |  | Diammonium Glycyrrhizinate | Indigo | Camphor | Oleanolic Acid | Glycyrrhizic acid | DL-Menthol | Bergenin | | Ferulic Acid | | Succinic acid | Paeonol |  |
| d | Empty | | T1238 | T0595 | T0966 | T2142 | T1034 | T1039 | T1040 | | T1045 | | T1051 | T1062 | Empty |
|  |  |  | Daidzein | Naringin | Sennoside A | Yohimbine hydrochloride | Docetaxel | Choline chloride | Vitamin B12 | | Trimipramine maleate | | Retinoic acid | Capsaicin |  |
| e | Empty | | T1285 | T1313 | T1314 | T1317 | T1323 | T1334 | T1231 | | T2907 | | T2806 | T2782 | Empty |
|  |  |  | Anethole trithione | Nitrofurantoin | Triclosan | Cinchonidine | Artemether | Hydroquinidine | Pilocarpine nitrate | | Tanshinone I | | Lappaconitine | Catharanthine |  |
| f | Empty | | T2921 | T2764 | T2793 | T2735 | T2965 | T2963 | T2136 | | T0646 | | T2565 | T0725 | Empty |
|  |  |  | Sinomenine hydrochloride | (S)-10-Hydroxycamptothecin | Tetrahydropalmatine | 5-Acetylsalicylic acid | Deoxycholic acid | Cholic Acid | Geniposidic Acid | | 5-Aminosalicylic Acid | | Hesperetin | Isoliquiritigenin |  |
| g | Empty | | T0775 | T0772 | T0759 | T0746 | T0743 | T0728 | T0877 | | T0878 | | T0879 | T0739 | Empty |
|  |  |  | Coumarin | Troxerutin | Monobenzone | Orotic acid | DL-Carnitine | Ethosuximide | Gallic acid | | Diosmin | | Niacin | Guaifenesin |  |
| h | Empty | | T2212 | T0649 | T3004 | T3000 | T3002 | T2995 | T2996 | | T2953 | | T2968 | T2745 | Empty |
|  |  |  | Geniposide | Salicylic acid | Batilol | Magnolol | Piperine | Chrysophanol | Tetrandrine | | Ligustrazine hydrochloride | | Hyodeoxycholic acid | 18α-Glycyrrhetinic acid |  |
| Plate layout:PHD147296 | | | | | | | | | | | | | | | |
|  | 1 | | 2 | 3 | 4 | 5 | 6 | 7 | 8 | | 9 | | 10 | 11 | 12 |
| a | Empty | | T2775 | T2898 | T2827 | T2807 | T2845 | T2815 | T2754 | | T2858 | | T2870 | T0795 | Empty |
|  |  |  | Baicalin | Andrographolide | Asiatic acid | Caffeic Acid | Imperatorin | Puerarin | Oxymatrine | | Baicalein | | Matrine | Rutin |  |
| b | Empty | | T0822 | T2563 | T0962 | T0937 | T1391 | T1396 | T1407 | | T1431 | | T1436 | T1529 | Empty |
|  |  |  | Catechin | Acetyl-L-carnitine hydrochloride | L-Hyoscyamine | Riboflavin | 4-Methylumbelliferone | Bemegride | (-)-Menthol | | Khellin | | 4-Aminohippuric Acid | Miglitol |  |
| c | Empty | | T1537 | T1558 | T1330 | T1737 | T2742 | T2144 | T2183 | | T1571 | | T1589 | T1591 | Empty |
|  |  |  | Rapamycin | Resveratrol | Acitretin | Genistein | Aloin | Tacrolimus | Scopolamine butylbromide | | Estriol | | D-Cycloserine | Ancitabine hydrochloride |  |
| d | Empty | | T1653 | T1659 | T1660 | T2543 | T2920 | T2984 | T2917 | | T2720 | | T2909 | T2851 | Empty |
|  |  |  | Liothyronine | Melatonin | Silibinin | Cabazitaxel | Berbamine dihydrochloride | Scopoletin | Tetrahydropalmatine hydrochloride | | Ginsenoside Rc | | Fraxetin | Daphnetin |  |
| e | Empty | | T2804 | T2901 | T2942 | T2855 | T2916 | T2727 | T2850 | | T2759 | | T3027 | T3026 | Empty |
|  |  |  | Panaxatriol | Daidzin | Psoralen | Icariin | Patchouli alcohol | Salvianolic acid B | Bicuculline | | Loganin | | Sodium Aescinate | (-)-Huperzine A |  |
| f | Empty | | T2823 | T2736 | T2722 | T2966 | T2179 | T3121 | T3136 | | T6670 | | T2149 | T1287 | Empty |
|  |  |  | Crocin | Sodium Demethylcantharidate | Ligustrazine | Beta-Sitosterol | Triptolide | Betulin | Methylcobalamin | | Silymarin | | Vinburnine | Synephrine |  |
| g | Empty | | T1286 | T2832 | T2756 | T2946 | T2854 | T1400 | T0968 | | T0970 | | T1670 | T1882 | Empty |
|  |  |  | Vincamine | Isocorydine | Swertiamarin | Tanshinone IIA sulfonate sodium | Phillyrin | Demeclocycline hydrochloride | Paclitaxel | | Racanisodamine | | Lanatoside C | Meisoindigo |  |
| h | Empty | | T3159 | T1183 | T3237 | T3233 | T3232 | T3273 | T3243 | | T3253 | | T6717 | T3339 | Empty |
|  |  |  | Guaiazulene | Retinol | Lecithin | Sophoricoside | Higenamine hydrochloride | Bifendate | Betaine | | Castor oil | | Vanillin | Sophoridine |  |
| Plate layout:PHD147297 | | | | | | | | | | | | | | | |
|  | | 1 | 2 | 3 | 4 | 5 | 6 | 7 | | 8 | | 9 | 10 | 11 | 12 |
| a | Empty | | T3327 | T6473 | T3362 | T3402 | T3380 | T3428 | T3667 | | T6293 | | T3698 | T6485 | Empty |
|  |  |  | Artemotil | Dioscin | Eupatilin | 20(S)-Ginsenoside Rg3 | Homoharringtonine | Helicid | Kalii Dehydrographolidi Succinas | | Mycophenolate Mofetil | | alpha-Asarone | Embelin |  |
| b | Empty | | T3501 | T3718 | T3823 | T6518 | T3529 | T6702 | T3763 | | T3824 | | T6430 | T6316 | Empty |
|  |  |  | Colistimethate Sodium | Santonin | D-Pinitol | Gossypol | Bestatin hydrochloride | Terbinafine | Fumaric acid | | Jaceosidin | | Calcifediol | Calcitriol |  |
| c | Empty | | T6560 | T3S1866 | T4S0797 | T6795 | T5S0802 | T5S1058 | T3S0807 | | T6169 | | T2A2481 | T4S1619 | Empty |
|  |  |  | Lappaconitine hydrobromide | Propyl gallate | Berberine | Carbidopa | Palmatine | Triptonide | Berbamine | | Indirubin | | Taurochenodeoxycholic Acid | L-Hyoscyamine sulfate |  |
| d | Empty | | T5S0814 | T2S0257 | T6723 | T3S0717 | T5S1891 | T7004 | T7056 | | T4333 | | T6873 | T6384 | Empty |
|  |  |  | Berberine hydrogen sulphate | Dehydroandrographolide succinate | Voglibose | Thymol | Flaconitine | Tubercidin | Dronedarone | | Quinine dihydrochloride | | Lauric Acid | Ammonium Glycyrrhizinate |  |
| e | Empty | | T4558 | T4550 | T4514 | T4590 | T4749 | T4874 | T4923 | | T4868 | | T4723 | T5077 | Empty |
|  |  |  | Trioxsalen | Ajmaline | N-Acetyl-D-Glucosamine | (+)-Kavain | Squalene | Thiamine pyrophosphate | 7-Dehydrocholesterol | | gamma-Linolenic acid | | D-Tagatose | Deoxycholic acid sodium salt |  |
| f | Empty | | T5067 | T5059 | T5014 | T5026 | T5040 | T5666 | T5723 | | T6805 | | T6460 | T5656 | Empty |
|  |  |  | Flavin adenine dinucleotide disodium salt | L-Tartaric acid | Prostaglandin E2 | Oxytocin | Medroxyprogesterone | Dipotassium glycyrrhizinate | Menthol | | Choline bitartrate | | Cysteamine hydrochloride | (±)-Norcantharidin |  |
| g | Empty | | T5940 | T7106 | T7094 | T7939 | T6841 | T4537 | T6751 | | T5041 | | T7938 | T2S0342 | Empty |
|  |  |  | Proxyphylline | Menaquinone-4 | Levomefolic Acid | Fingolimod | Fumagillin | Cholic acid sodium | Acetylcholine iodide | | Faropenem sodium | | Quinidine | Armillarisin A |  |
| h | Empty | | T3S2100 | T8149 | T4S1725 | T8270 | T6475 | T6631 | T6752 | | T8183 | | T6648 | T17199 | Empty |
|  |  |  | (-)-Securinine | Dobutamine hydrochloride | Galanthamine | Deserpidine | (±)-Carnitine chloride | Quinine hydrochloride dihydrate | S-Adenosyl-L-methionine disulfate tosylate | | Deslanoside | | Rotundine | Udenafil |  |
| Plate layout:PHD147298 | | | | | | | | | | | | | | | |
|  | | 1 | 2 | 3 | 4 | 5 | 6 | 7 | | 8 | | 9 | 10 | 11 | 12 |
| a | Empty | | T14932 | T8689 | T8709 | T8286 | T20884 | T14291 | T14865 | | T14128 | | T8516 | TQ0197 | Empty |
|  |  |  | Ceruletide | Chloroquine | Nandrolone phenylpropionate | Harringtonine | Hydroxyzine | Anisindione | Carboprost tromethamine | | Adenosylcobalamin | | Levomenol | Acenocoumarol |  |
| b | Empty | | T9171 | T12095 | T13923 | T29078 | T21532 | T1662 | T0501 | | T1035 | | T0769 | T0607 | Empty |
|  |  |  | Methenolone enanthate | Monomethyl fumarate | Thiamine nitrate | Ursodeoxycholic acid sodium | Anisodamine | 5-Aminolevulinic acid hydrochloride | Mequinol | | Hesperidin | | Aceglutamide | Dihydroartemisinin |  |
| c | Empty | | T2999 | T2729 | T2768 | T2762 | T4S0647 | T6369 | T2721 | | T0625 | | T2843 | T1611 | Empty |
|  |  |  | Gastrodin | Scoparone | Saikosaponin A | Kasugamycin hydrochloride | Butylphthalide | Adenine hemisulfate | Digitonin | | Theobromine | | Aloe emodin | Isotretinoin |  |
| d | Empty | | T5292 | T5557 | T7060 | T6431 | T1331 | T4987 | T6272 | | T7908 | | T0022 | Empty | Empty |
|  |  |  | N-Acetyl-L-tyrosine | Propylparaben | Amantadine | Levoleucovorin Calcium | Riboflavin phosphate sodium | NSC 42196 | Fosbretabulin Disodium | | DL-Methionine Methylsulfonium Chloride | | Taurine |  |  |
| e | Empty | | Empty | Empty | Empty | Empty | Empty | Empty | Empty | | Empty | | Empty | Empty | Empty |
| f | Empty | | Empty | Empty | Empty | Empty | Empty | Empty | Empty | | Empty | | Empty | Empty | Empty |
| g | Empty | | Empty | Empty | Empty | Empty | Empty | Empty | Empty | | Empty | | Empty | Empty | Empty |
| h | Empty | | Empty | Empty | Empty | Empty | Empty | Empty | Empty | | Empty | | Empty | Empty | Empty |

**Supplementary Table 1.** A custom compound library of 279 approved drugs.
