# Supplementary material for: Medroxyprogesterone promotes neuronal survival after cerebral ischemic stroke by inhibiting PARthanatos
Source: Front Pharmacol. 2025 Feb 13;16:1487436. doi: 10.3389/fphar.2025.1487436 (PMC11865058; doi:10.3389/fphar.2025.1487436)
Supplement: Supplementary file 1 [file DataSheet1.zip › Table 2.docx]

| **SwissTargetPrediction** | | | | | | |
| --- | --- | --- | --- | --- | --- | --- |
| **Target** | **Common name** | **Uniprot ID** | **ChEMBL ID** | **Target Class** | **Probability*** | **Known actives (3D/2D)** |
| Androgen Receptor (by homology) | AR | P10275 | CHEMBL1871 | Nuclear receptor | 0.872151955 | 187 / 138 |
| Glucocorticoid receptor | NR3C1 | P04150 | CHEMBL2034 | Nuclear receptor | 0.872151955 | 188 / 43 |
| Dopamine transporter | SLC6A3 | Q01959 | CHEMBL238 | Electrochemical transporter | 0.872151955 | 205 / 1 |
| Corticosteroid binding globulin | SERPINA6 | P08185 | CHEMBL2421 | Secreted protein | 0.872151955 | 9 / 23 |
| Adenosine A3 receptor | ADORA3 | P0DMS8 | CHEMBL256 | Family A G protein-coupled receptor | 0.872151955 | 19 / 3 |
| Testis-specific androgen-binding protein | SHBG | P04278 | CHEMBL3305 | Secreted protein | 0.872151955 | 45 / 54 |
| MAP kinase ERK1 | MAPK3 | P27361 | CHEMBL3385 | Kinase | 0.872151955 | 5 / 1 |
| Cytochrome P450 17A1 | CYP17A1 | P05093 | CHEMBL3522 | Cytochrome P450 | 0.741353386 | 54 / 48 |
| Progesterone receptor | PGR | P06401 | CHEMBL208 | Nuclear receptor | 0.724592707 | 92 / 50 |
| Cytochrome P450 19A1 | CYP19A1 | P11511 | CHEMBL1978 | Cytochrome P450 | 0.632916206 | 109 / 332 |
| Sigma opioid receptor | SIGMAR1 | Q99720 | CHEMBL287 | Membrane receptor | 0.373317398 | 168 / 4 |
| Niemann-Pick C1-like protein 1 | NPC1L1 | Q9UHC9 | CHEMBL2027 | Other membrane protein | 0.289727852 | 0 / 12 |
| 11-beta-hydroxysteroid dehydrogenase 2 | HSD11B2 | P80365 | CHEMBL3746 | Enzyme | 0.281242088 | 7 / 26 |
| 11-beta-hydroxysteroid dehydrogenase 1 | HSD11B1 | P28845 | CHEMBL4235 | Enzyme | 0.281242088 | 251 / 43 |
| Mineralocorticoid receptor | NR3C2 | P08235 | CHEMBL1994 | Nuclear receptor | 0.239365343 | 40 / 28 |
| Dual specificity phosphatase Cdc25A | CDC25A | P30304 | CHEMBL3775 | Phosphatase | 0.21440508 | 11 / 16 |
| Protein farnesyltransferase | FNTA FNTB | P49354 P49356 | CHEMBL2094108 | Enzyme | 0.164184052 | 50 / 12 |
| Protein kinase C eta | PRKCH | P24723 | CHEMBL3616 | Kinase | 0.164184052 | 10 / 1 |
| Prostaglandin E synthase | PTGES | O14684 | CHEMBL5658 | Enzyme | 0.164184052 | 22 / 13 |
| Protein-tyrosine phosphatase 2C | PTPN11 | Q06124 | CHEMBL3864 | Phosphatase | 0.147460274 | 0 / 2 |
| Aldo-keto reductase family 1 member B10 | AKR1B10 | O60218 | CHEMBL5983 | Enzyme | 0.147460274 | 0 / 7 |
| Estrogen receptor beta | ESR2 | Q92731 | CHEMBL242 | Nuclear receptor | 0.147460274 | 44 / 47 |
| Serotonin transporter | SLC6A4 | P31645 | CHEMBL228 | Electrochemical transporter | 0.130706653 | 285 / 5 |
| T-cell protein-tyrosine phosphatase | PTPN2 | P17706 | CHEMBL3807 | Phosphatase | 0.122339194 | 0 / 20 |
| Steroid 5-alpha-reductase 2 | SRD5A2 | P31213 | CHEMBL1856 | Oxidoreductase | 0.122339194 | 18 / 43 |
| Indoleamine 2,3-dioxygenase | IDO1 | P14902 | CHEMBL4685 | Enzyme | 0.122339194 | 9 / 1 |
| Beta-secretase 1 | BACE1 | P56817 | CHEMBL4822 | Protease | 0.122339194 | 176 / 2 |
| Estradiol 17-beta-dehydrogenase 3 | HSD17B3 | P37058 | CHEMBL4234 | Enzyme | 0.113979815 | 13 / 12 |
| Steroid 5-alpha-reductase 1 | SRD5A1 | P18405 | CHEMBL1787 | Oxidoreductase | 0.113979815 | 19 / 10 |
| Arachidonate 5-lipoxygenase | ALOX5 | P09917 | CHEMBL215 | Oxidoreductase | 0.10560828 | 49 / 41 |
| Estrogen receptor alpha | ESR1 | P03372 | CHEMBL206 | Nuclear receptor | 0.10560828 | 39 / 39 |
| Nitric oxide synthase, inducible | NOS2 | P35228 | CHEMBL4481 | Enzyme | 0.10560828 | 14 / 20 |
| Butyrylcholinesterase | BCHE | P06276 | CHEMBL1914 | Hydrolase | 0.10560828 | 14 / 2 |
| Protein-tyrosine phosphatase 1B | PTPN1 | P18031 | CHEMBL335 | Phosphatase | 0.10560828 | 10 / 60 |
| Fatty acid-binding protein, liver (by homology) | FABP1 | P07148 | CHEMBL5421 | Fatty acid binding protein family | 0.10560828 | 0 / 3 |
| Squalene synthetase | FDFT1 | P37268 | CHEMBL3338 | Enzyme | 0.097239989 | 10 / 1 |
| Glucose-6-phosphate 1-dehydrogenase | G6PD | P11413 | CHEMBL5347 | Enzyme | 0.097239989 | 2 / 7 |
| DNA polymerase beta (by homology) | POLB | P06746 | CHEMBL2392 | Enzyme | 0.097239989 | 1 / 10 |
| Acyl coenzyme A:cholesterol acyltransferase | CES1 | P23141 | CHEMBL2265 | Enzyme | 0.097239989 | 0 / 5 |
| Protein-tyrosine phosphatase 1C | PTPN6 | P29350 | CHEMBL3166 | Phosphatase | 0.097239989 | 0 / 1 |
| HMG-CoA reductase | HMGCR | P04035 | CHEMBL402 | Oxidoreductase | 0.097239989 | 131 / 27 |
| Pregnane X receptor | NR1I2 | O75469 | CHEMBL3401 | Nuclear receptor | 0.097239989 | 5 / 3 |
| Nuclear receptor ROR-alpha | RORA | P35398 | CHEMBL5868 | Nuclear receptor | 0.097239989 | 1 / 4 |
| Fatty acid binding protein adipocyte | FABP4 | P15090 | CHEMBL2083 | Fatty acid binding protein family | 0.097239989 | 0 / 3 |
| Fatty acid binding protein muscle | FABP3 | P05413 | CHEMBL3344 | Fatty acid binding protein family | 0.097239989 | 0 / 4 |
| Fatty acid binding protein epidermal | FABP5 | Q01469 | CHEMBL3674 | Fatty acid binding protein family | 0.097239989 | 0 / 1 |
| Peroxisome proliferator-activated receptor delta | PPARD | Q03181 | CHEMBL3979 | Nuclear receptor | 0.097239989 | 0 / 9 |
| Peroxisome proliferator-activated receptor gamma | PPARG | P37231 | CHEMBL235 | Nuclear receptor | 0.097239989 | 6 / 24 |
| Interleukin-6 | IL6 | P05231 | CHEMBL1795129 | Secreted protein | 0.097239989 | 0 / 1 |
| Glutamine synthetase | GLUL | P15104 | CHEMBL4612 | Ligase | 0.097239989 | 0 / 1 |
| Estradiol 17-beta-dehydrogenase 2 | HSD17B2 | P37059 | CHEMBL2789 | Enzyme | 0.097239989 | 65 / 2 |
| Aldo-keto-reductase family 1 member C3 | AKR1C3 | P42330 | CHEMBL4681 | Enzyme | 0.097239989 | 11 / 1 |
| Acyl-CoA desaturase | SCD | O00767 | CHEMBL5555 | Enzyme | 0.097239989 | 0 / 1 |
| Receptor-type tyrosine-protein phosphatase F (LAR) | PTPRF | P10586 | CHEMBL3521 | Membrane receptor | 0.097239989 | 0 / 1 |
| Phospholipase A2 group 1B | PLA2G1B | P04054 | CHEMBL4426 | Enzyme | 0.097239989 | 0 / 1 |
| Low molecular weight phosphotyrosine protein phosphatase | ACP1 | P24666 | CHEMBL4903 | Phosphatase | 0.097239989 | 0 / 1 |
| Carboxylesterase 2 | CES2 | O00748 | CHEMBL3180 | Enzyme | 0.097239989 | 2 / 15 |
| C-C chemokine receptor type 1 | CCR1 | P32246 | CHEMBL2413 | Family A G protein-coupled receptor | 0.097239989 | 101 / 0 |
| Anandamide amidohydrolase | FAAH | O00519 | CHEMBL2243 | Enzyme | 0.097239989 | 15 / 14 |
| Aldo-keto reductase family 1 member C2 | AKR1C2 | P52895 | CHEMBL5847 | Enzyme | 0.097239989 | 0 / 1 |
| Aldo-keto reductase family 1 member C1 | AKR1C1 | Q04828 | CHEMBL5905 | Enzyme | 0.097239989 | 0 / 1 |
| Nuclear receptor subfamily 1 group I member 3 (by homology) | NR1I3 | Q14994 | CHEMBL5503 | Nuclear receptor | 0.097239989 | 1 / 2 |
| Cytochrome P450 11B1 | CYP11B1 | P15538 | CHEMBL1908 | Cytochrome P450 | 0.097239989 | 19 / 0 |
| Cytochrome P450 11B2 | CYP11B2 | P19099 | CHEMBL2722 | Cytochrome P450 | 0.097239989 | 19 / 0 |
| HERG | KCNH2 | Q12809 | CHEMBL240 | Voltage-gated ion channel | 0.097239989 | 41 / 0 |
| Prolyl endopeptidase | PREP | P48147 | CHEMBL3202 | Protease | 0.097239989 | 1 / 2 |
| Cytochrome P450 51 | CYP51A1 | Q16850 | CHEMBL3849 | Cytochrome P450 | 0.097239989 | 12 / 2 |
| Cytochrome P450 2C19 | CYP2C19 | P33261 | CHEMBL3622 | Cytochrome P450 | 0.097239989 | 6 / 6 |
| Gamma-secretase | PSEN2 PSENEN NCSTN APH1A PSEN1 APH1B | P49810 Q9NZ42 Q92542 Q96BI3 P49768 Q8WW43 | CHEMBL2094135 | Protease | 0.097239989 | 133 / 0 |
| Cyclooxygenase-1 | PTGS1 | P23219 | CHEMBL221 | Oxidoreductase | 0.097239989 | 8 / 2 |
| Neuropeptide Y receptor type 5 | NPY5R | Q15761 | CHEMBL4561 | Family A G protein-coupled receptor | 0.097239989 | 47 / 0 |
| CD81 antigen | CD81 | P60033 | CHEMBL1075180 | Surface antigen | 0.097239989 | 0 / 1 |
| Tyrosine-protein kinase JAK3 | JAK3 | P52333 | CHEMBL2148 | Kinase | 0.097239989 | 88 / 0 |
| Tyrosine-protein kinase JAK1 | JAK1 | P23458 | CHEMBL2835 | Kinase | 0.097239989 | 93 / 0 |
| Tyrosine-protein kinase TYK2 | TYK2 | P29597 | CHEMBL3553 | Kinase | 0.097239989 | 37 / 0 |
| Geranylgeranyl transferase type I beta subunit | PGGT1B | P53609 | CHEMBL4135 | Enzyme | 0.097239989 | 10 / 0 |
| LXR-alpha | NR1H3 | Q13133 | CHEMBL2808 | Nuclear receptor | 0.097239989 | 5 / 20 |
| Vanilloid receptor | TRPV1 | Q8NER1 | CHEMBL4794 | Voltage-gated ion channel | 0.097239989 | 59 / 2 |
| C-C chemokine receptor type 5 | CCR5 | P51681 | CHEMBL274 | Family A G protein-coupled receptor | 0.097239989 | 7 / 1 |
| Cyclooxygenase-2 | PTGS2 | P35354 | CHEMBL230 | Oxidoreductase | 0.097239989 | 47 / 5 |
| Cannabinoid receptor 1 (by homology) | CNR1 | P21554 | CHEMBL218 | Family A G protein-coupled receptor | 0.097239989 | 149 / 8 |
| Muscarinic acetylcholine receptor M2 | CHRM2 | P08172 | CHEMBL211 | Family A G protein-coupled receptor | 0.097239989 | 66 / 1 |
| Glucagon receptor | GCGR | P47871 | CHEMBL1985 | Family B G protein-coupled receptor | 0.097239989 | 20 / 0 |
| Liver glycogen phosphorylase | PYGL | P06737 | CHEMBL2568 | Enzyme | 0.097239989 | 43 / 0 |
| Serine/threonine-protein kinase PIM1 | PIM1 | P11309 | CHEMBL2147 | Kinase | 0.097239989 | 32 / 0 |
| Serine/threonine-protein kinase PIM3 | PIM3 | Q86V86 | CHEMBL5407 | Kinase | 0.097239989 | 11 / 0 |
| Mitogen-activated protein kinase kinase kinase 14 | MAP3K14 | Q99558 | CHEMBL5888 | Kinase | 0.097239989 | 3 / 0 |
| Nuclear receptor ROR-gamma | RORC | P51449 | CHEMBL1741186 | Nuclear receptor | 0.097239989 | 28 / 10 |
| Tyrosine-protein kinase JAK2 | JAK2 | O60674 | CHEMBL2971 | Kinase | 0.097239989 | 103 / 0 |
| C5a anaphylatoxin chemotactic receptor | C5AR1 | P21730 | CHEMBL2373 | Family A G protein-coupled receptor | 0.097239989 | 30 / 0 |
| Phosphodiesterase 4D | PDE4D | Q08499 | CHEMBL288 | Phosphodiesterase | 0.097239989 | 4 / 1 |
| Cyclin-dependent kinase 1/cyclin B | CCNB3 CDK1 CCNB1 CCNB2 | Q8WWL7 P06493 P14635 O95067 | CHEMBL2094127 | Other cytosolic protein | 0.097239989 | 6 / 0 |
| Cyclin-dependent kinase 1 | CDK1 | P06493 | CHEMBL308 | Kinase | 0.097239989 | 42 / 0 |
| Telomerase reverse transcriptase | TERT | O14746 | CHEMBL2916 | Enzyme | 0.097239989 | 3 / 3 |
| Dopamine D2 receptor | DRD2 | P14416 | CHEMBL217 | Family A G protein-coupled receptor | 0.097239989 | 145 / 0 |
| Dopamine D4 receptor | DRD4 | P21917 | CHEMBL219 | Family A G protein-coupled receptor | 0.097239989 | 46 / 0 |
| ADAM17 | ADAM17 | P78536 | CHEMBL3706 | Protease | 0.097239989 | 29 / 1 |
| Monoamine oxidase A (by homology) | MAOA | P21397 | CHEMBL1951 | Oxidoreductase | 0.097239989 | 14 / 0 |
| Monoamine oxidase B (by homology) | MAOB | P27338 | CHEMBL2039 | Oxidoreductase | 0.097239989 | 11 / 0 |
| Protein kinase C gamma | PRKCG | P05129 | CHEMBL2938 | Kinase | 0.097239989 | 18 / 0 |

**Supplementary Table 2.** Prediction of medroxyprogesterone target using SwissTargetPredicition.
